# Comprehensive profiling of 1015 patients’ exomes reveals genomic-clinical associations in colorectal cancer

**DOI:** 10.1038/s41467-022-30062-8

**Published:** 2022-04-29

**Authors:** Qi Zhao, Feng Wang, Yan-Xing Chen, Shifu Chen, Yi-Chen Yao, Zhao-Lei Zeng, Teng-Jia Jiang, Ying-Nan Wang, Chen-Yi Wu, Ying Jing, You-Sheng Huang, Jing Zhang, Zi-Xian Wang, Ming-Ming He, Heng-Ying Pu, Zong-Jiong Mai, Qi-Nian Wu, Renwen Long, Xiaoni Zhang, Tanxiao Huang, Mingyan Xu, Miao-Zheng Qiu, Hui-Yan Luo, Yu-Hong Li, Dong-Shen Zhang, Wei-Hua Jia, Gong Chen, Pei-Rong Ding, Li-Ren Li, Zheng-Hai Lu, Zhi-Zhong Pan, Rui-Hua Xu

**Affiliations:** 1 State Key Laboratory of Oncology in South China, Collaborative Innovation Center for Cancer Medicine, Sun Yat-sen University Cancer Center, Sun Yat-sen University, Guangzhou, 510060 P. R. China; 2 HaploX Biotechnology, Co., Ltd., 8th floor, Auto Electric Power Building, Songpingshan Road, Nanshan District, Shenzhen, Guangdong 518057 P. R. China; 3 Research Unit of Precision Diagnosis and Treatment for Gastrointestinal Cancer, Chinese Academy of Medical Sciences, Guangzhou, 510060 P. R. China

**Keywords:** Colorectal cancer, Cancer genomics, Cancer genomics, Tumour biomarkers, Tumour heterogeneity

## Abstract

The genetic basis of colorectal cancer (CRC) and its clinical associations remain poorly understood due to limited samples or targeted genes in current studies. Here, we perform ultradeep whole-exome sequencing on 1015 patients with CRC as part of the ChangKang Project. We identify 46 high-confident significantly mutated genes, 8 of which mutate in 14.9% of patients: *LYST*, *DAPK1*, *CR2*, *KIF16B*, *NPIPB15*, *SYTL2*, *ZNF91*, and *KIAA0586*. With an unsupervised clustering algorithm, we propose a subtyping strategy that classisfies CRC patients into four genomic subtypes with distinct clinical characteristics, including hypermutated, chromosome instability with high risk, chromosome instability with low risk, and genome stability. Analysis of immunogenicity uncover the association of immunogenicity reduction with genomic subtypes and poor prognosis in CRC. Moreover, we find that mitochondrial DNA copy number is an independent factor for predicting the survival outcome of CRCs. Overall, our results provide CRC-related molecular features for clinical practice and a valuable resource for translational research.

## Abstract (web summary)

The ChangKang (Heathy Bowel) project was established to collect molecular and clinical information of a thousand Chinese colorectal cancer patients. Here, the authors present the genomic landscape of the ChangKang cohort and find a subgroup of patients defined by abnormal mitochondrial copy numbers.

## Introduction

Colorectal cancer (CRC) is the third most common cancer worldwide, accounting for an estimated 1.87 million new cases and more than 910,000 deaths in 2020^1^. It was one of the first genomic-defined human cancers with extremely high heterogeneity^2^. The current known risk factors for CRC include processed meat consumption, alcohol intake, obesity, inflammatory bowel disease (IBD), a family history of CRC, CRC-predisposing genetic variants, et al.^3,4^. The establishment of representative genomic landscapes of CRC is fundamental to downstream transitional research and the further development of novel therapeutics.

The recent large genomic projects presented by The Cancer Genome Atlas (TCGA) and other groups adopted high-throughput technology including whole genome sequencing (WGS), whole-exome sequencing^5^, and targeted sequencing to elaborate the genetic landscape of CRC and reported several significantly mutated genes (SMGs) and recurrent copy number alterations(CNAs)^6–8^. However, these genomic projects/studies were limited by small sample sizes, analysis of patients mainly from the Western population, insufficient patients’ treatment/outcome information, and limited protein-coding genes in panel-based sequencing. In addition, the mitochondrial genome and its association with genomic alterations as well as clinical characteristics in CRC are still elusive.

To address these issues, we launched the ChangKang (Heathy Bowel) project in 2017, aiming to establish a large genomic and clinical database including a thousand patients with CRC from the Chinese population to provide information for early screening and diagnosis, prognostic evaluation, postoperative recurrence monitoring, individualized medication, efficacy evaluation, and drug resistance analysis for patients with CRC.

In this study, we examined the genomic alterations in a large CRC cohort and revealed their clinical relevance with a series of well-established bioinformatics approaches. Collectively, the results of this study will provide rich resources to identify promising biomarkers, therapeutic targets, molecular subtypes, and prognostic assessments for CRC.

## Results

### Samples and clinical data

**Fig. 1:**
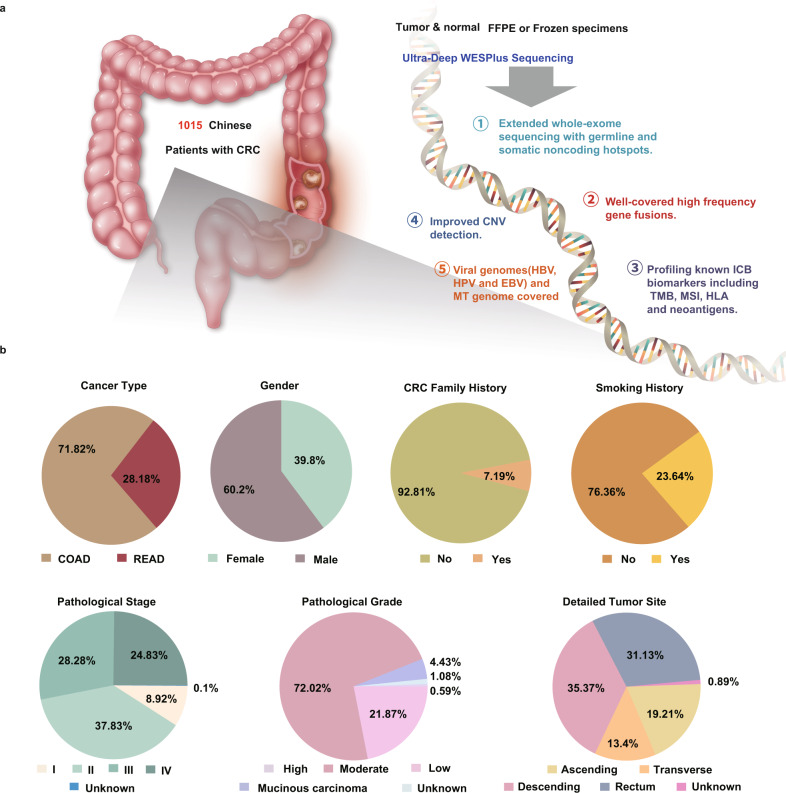
Schematic diagram of the ChangKang study. **a** Sample collection workflow and core features of the sequencing platform; **b** Summarized characteristics of 1015 patients analyzed in this study.

The whole exomes of 1015 CRC and nonneoplastic tissues from each patient were sequenced and analyzed by using HapOnco WESplus, a capture-based next-generation sequencing product with improved sequencing depth that can detect mutations and copy-number alterations at the whole-exome level, three virus sequences, whole mitochondrial genomes, and alterations for predicting responses to immune checkpoint blockade treatments (Fig. 1a). All samples were collected retrospectively under institutional review board approval (Methods), and a detailed flowchart is presented in Supplementary Fig. 1a. The clinicopathological characteristics of this cohort are summarized in Table 1 and Fig. 1b. The median age at diagnosis was 59 years (range, 18 to 88), and 39.8% of the patients were female. In total, 72.41% of patients in the cohort had colon adenocarcinoma (COAD), and the remainder (27.59%) had rectal adenocarcinoma (READ). A family history of CRC was reported in 7.19% of patients, while a smoking history was reported in 23.64%. Approximately one-third of the patients (31.5%) had right-sided CRC (from the cecum to the splenic flexure), and the other two-thirds (68.5%) had left-sided CRC (from the splenic flexure to the rectum). The proportions of patients classified as having American Joint Committee on Cancer (AJCC) stage I, II, III, and IV diseases were 8.88%, 38.44%, 28%, and 24.59%, respectively. A total of 4.44% of the CRCs were defined as mucinous carcinoma, while 94.67% were defined as adenocarcinoma. The median follow-up time of the CRC cohort was 68.8 months (95% CI, 67.7 to 70.4), while the median overall survival (OS) was not reached. Compared with the TCGA cohort (Supplementary Fig. 1b), our cohort had a higher fraction of metastatic disease, a higher fraction of READ, a larger proportion of males, more left-sided primary tumors (all *P* < 0.001), younger ages at onset, a lower mucinous carcinoma fraction and a much lower rate of missing clinical information (only one patient), suggesting distinct clinical characteristics and improved clinical data quality of our cohort.

**Table 1:** clinical characteristic of colorectal cancer patients in hypermutated and non-hypermutated group.

Features	Total	Hypermutated	Non-Hypermutated	*p*
	*N* = 1015	*N* = 72	*N* = 943	
**Cancer type,** ***n*** **(%)**				**<0.001**
COAD	729 (71.8)	71 (98.6)	658 (69.8)	
READ	286 (28.2)	1 (1.4)	285 (30.2)	
**Gender,** ***n*** **(%)**				0.04
Female	404 (39.8)	20 (27.8)	384 (40.7)	
Male	611 (60.2)	52 (72.2)	559 (59.3)	
**Marital status,** ***n*** **(%)**				1
Married	1014 (99.9)	72 (100.0)	942 (99.9)	
Unmarried	1 (0.1)	0 (0.0)	1 (0.1)	
**Height (mean** ± **SD)**	163.18 ± 7.69	164.71 ± 7.06	163.06 ± 7.73	0.08
**Weight (mean** ± **SD)**	59.16 ± 10.13	59.60 ± 9.57	59.13 ± 10.17	0.71
**Smoking,** ***n*** **(%)**				0.07
No	771 (76.3)	48 (66.7)	723 (77.0)	
Yes	240 (23.7)	24 (33.3)	216 (23.0)	
**Age (mean** ± **SD)**	57.57 ± 12.51	49.58 ± 14.03	58.18 ± 12.18	**<0.001**
**Family history,** ***n*** **(%)**				0.07
No	785 (77.3)	49 (68.1)	736 (78.0)	
Yes	230 (22.7)	23 (31.9)	207 (22.0)	
**CRC family history,** ***n*** **(%)**				**<0.001**
No	942 (92.8)	57 (79.2)	885 (93.8)	
Yes	73 (7.2)	15 (20.8)	58 (6.2)	
**Detailed tumor site,** ***n*** **(%)**				**<0.001**
Ascending	195 (19.2)	36 (50.0)	159 (16.9)	
Descending	359 (35.4)	12 (16.7)	347 (36.8)	
Rectum	316 (31.1)	1 (1.4)	315 (33.4)	
Transverse	136 (13.4)	21 (29.2)	115 (12.2)	
Unknown	9 (0.9)	2 (2.8)	7 (0.7)	
**Primary tumor location,** ***n*** **(%)**				**<0.001**
Left side	695 (68.5)	15 (20.8)	680 (72.1)	
Right side	320 (31.5)	57 (79.2)	263 (27.9)	
**Pathological,** ***n*** **(%)**				**<0.001**
High	6 (0.6)	1 (1.4)	5 (0.5)	
Low	222 (21.9)	29 (40.3)	193 (20.5)	
Moderately	731 (72.0)	35 (48.6)	696 (73.8)	
Mucinous carcinoma	45 (4.4)	7 (9.7)	38 (4.0)	
Unknown	11 (1.1)	0 (0.0)	11 (1.2)	
**Pathological stage,** ***n*** **(%)**				**<0.001**
I	91 (9.0)	4 (5.6)	87 (9.2)	
II	384 (37.8)	47 (65.3)	337 (35.7)	
III	287 (28.3)	15 (20.8)	272 (28.8)	
IV	252 (24.8)	6 (8.3)	246 (26.1)	
Unknown	1 (0.1)	0 (0.0)	1 (0.1)	

### Hypermutation phenotype of colorectal cancer

**Fig. 2:**
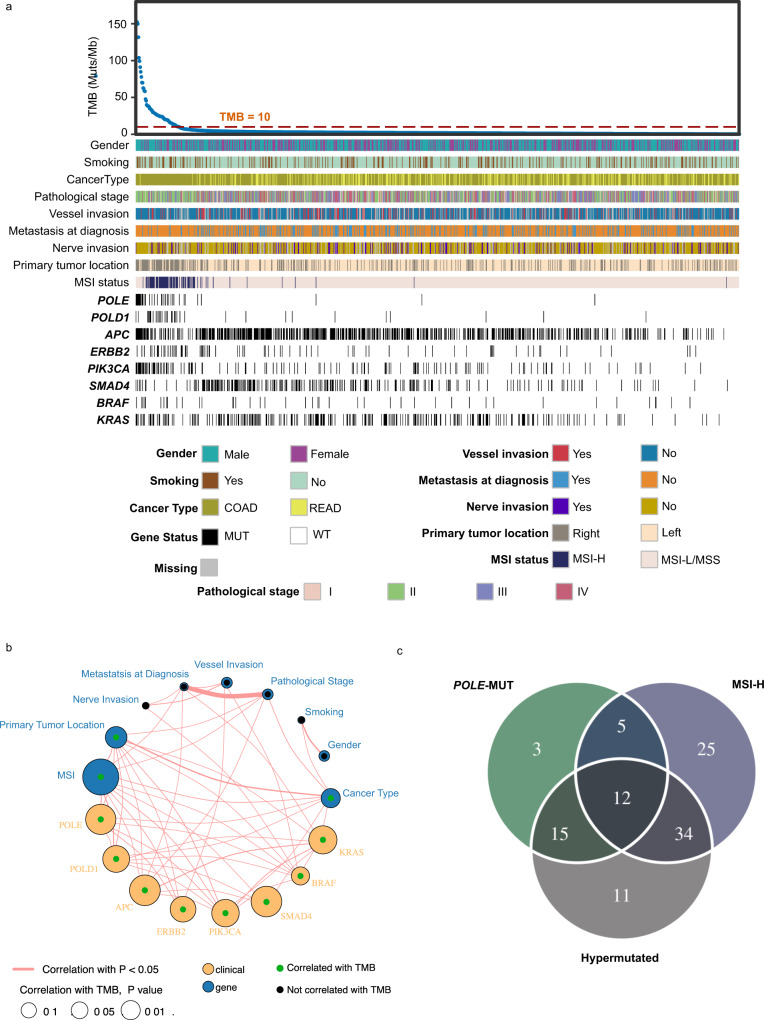
Hypermutated phenotype of CRC and its associations with clinical features. **a** Heatmap with multiple tracks presenting the different mutation profiles between the hypermutated and nonhypermutated groups. **b** Network plot presenting the correlation analysis of the clinical features and selected molecular events. The size of the node indicates the -log (*P*-value) calculated with the correlation test, while the circle in different colors represents the variable type (clinical or molecular event). **c** Overlap analysis of CRC with MSI-H, POLE mutations (nonsynonymous mutations), or the hypermutated phenotype (TMB > = 10).

The median depths of whole-exome sequencing coverage across all tumor and nonneoplastic tissues were 219×(43× to 661×) and 223×(100× to 665×), respectively, both of which were higher than those from the whole-exome dataset from the TCGA (Supplementary Fig. 2a, b and Supplementary Data 1). Notably, our dataset was more effective in identifying genetic alterations with low frequencies than that from the TCGA (Supplementary Fig. 2c). In this study, we identified a total of 277,090 nonsynonymous somatic variants (median 117.5, range 1–22760), which was significantly lower than that in the TCGA cohort but with larger variance (Supplementary Fig. 3). Tumor mutational burden (TMB) was determined sequentially (see Methods), with a median TMB of 1.74 (range 0–339.7). CRC is characterized by a high fraction of hypermutated phenotypes (Supplementary Data 2). Previous studies have shown that 15% to 20% of CRCs have an extremely high TMB defined by the numbers of nonsynonymous somatic mutations and small insertions/deletions (InDels) per mega base (Mb)^9–12^. By using a threshold of TMB ≥ 10 muts/Mb to define hypermutated cases^13^, we observed a relatively low (7.09%, 72/1015) hypermutated rate in our dataset compared with both the TCGA and MSKCC datasets. The microsatellite instability (MSI) status was determined with MSIsensor^14^. Next, we sought to link the hypermutated phenotype with clinical factors and several well-known CRC-related genomic alterations. As a result, in addition to *POLE* and the MSI status, we found that *POLD1*, *APC*, *ERBB2*, *PIK3CA*, *SMAD4*, *BRAF*, and *KRAS* were correlated with the hypermutated phenotype in CRC. Specifically, as shown in Fig. 2a, *POLE*, *POLD1*, and *PIK3CA* mutations were clearly enriched in the hypermutated group, while *SMAD4* alterations were more likely to occur in the nonhypermutated group.

Moreover, we observed differences in somatic copy-number variations (SCNVs) between the two groups. For the comparison of SCNVs at the gene level and lesion level, the hypermutated group had a significantly lower CNV burden than the nonhypermutated group (Supplementary Fig. 4), most of which were amplification events. This finding was also observed in a recent Japanese study^15^. Furthermore, the hypermutated phenotype was significantly associated with younger age, male sex, right-sided tumors, and pathological stage II (Fig. 2b and Table 1). Among all the hypermutated tumors, MSI-H and *POLE* nonsynonymous mutations were found in approximately 63.8% (46/72) and 37.5% (25/72) of patients, respectively. However, eleven hypermutated tumors could not be explained by MSI-H and *POLE* mutations (Fig. 2c).

### Identification of high-confidence SMGs (HC-SMGs) and mutation hotspots in CRC

**Fig. 3:**
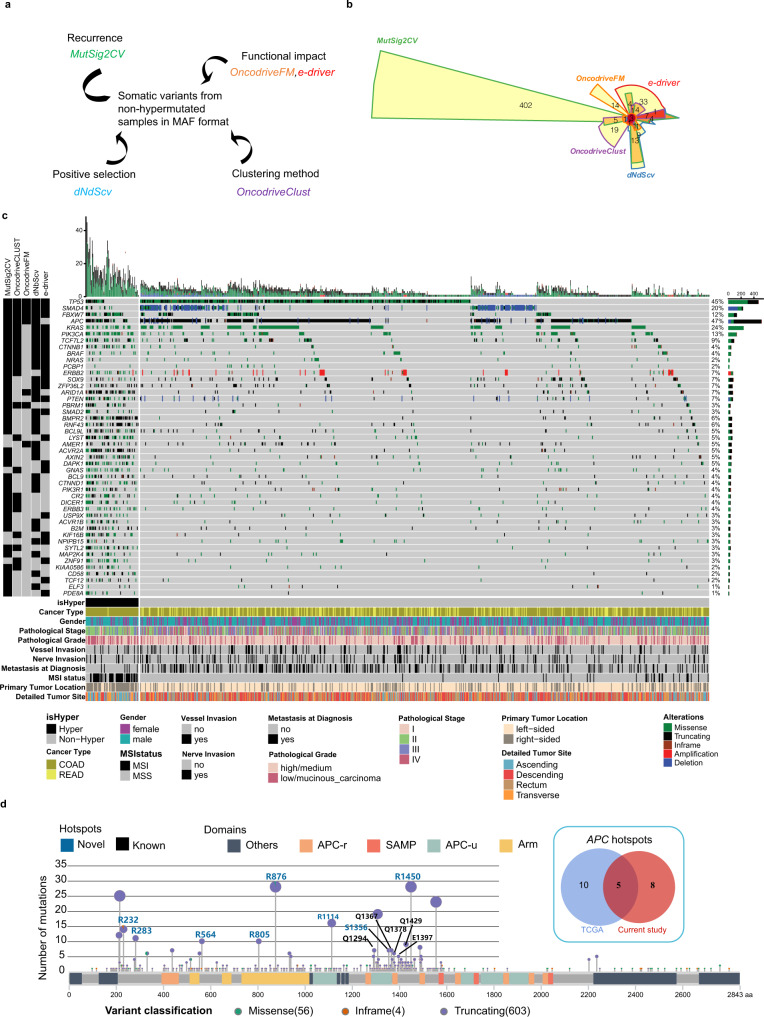
Identification of HC-SMGs in CRC. **a** Algorithms used to identify HC-SMGs; **b** Overlap analysis of the recurrent genes identified by each algorithm; **c** Significantly mutated genes identified in SYSUCC-CRC patients. **Top:** Bars represent the somatic mutation rate for CRC samples with different mutation types distinguished by color. **Bottom left:** Tools that were used to identify the significant genes. **Bottom right:** Mutation status of the significantly mutated genes in each CRC sample, identified by more than two tools, ranked by mutation frequency. **d** Lollipop plot show the distribution of identified hotspot in *APC* gene. The Venn diagram show the overlapped hotspot in *APC* between the current study and the TCGA cohort.

One of the major goals of cancer genomics research is to identify HC-SMGs, which are probably linked to tumorigenesis directly. Therefore, by examining somatic mutations in nonhypermutated patients, we applied an integrative screening approach to determine the HC-SMGs as follows: first, we applied several complementary SMG discovery tools to identify the candidate genes; second, only genes identified by more than one tool were considered HC-SMGs. Each tool identified the SMGs based on different features, including high recurrent mutations within a gene (MutSigCV), mutational clustering (OncodriveClust), heavy functional impact on gene function (OncodriveFM, e-driver), and positive selection on certain genes (dNdScv) (Fig. 3a). In our analysis, different methods identified distinct candidate gene lists (Fig. 3b). For example, MuSig2CV uniquely identified 402 more SMGs that were not identified by other tools.

By applying this strategy, we finally obtained 46 HC-SMGs (Fig. 3c). To confirm our findings, we annotated the HC-SMGs according to 5 databases and the literature: CGC^16^, IntOGen^17^, OncoKB^18^, TCGA, and Vogelstein et al.^19^ (Supplementary Data 3). By sorting the HC-SMGs based on the numbers of supported databases, we found that known CRC driver genes, such as *TP53, APC, KRAS, FBXW7*, and *CTNNB1*, ranked at the top of the HC-SMG list, suggesting that the SMGs identified in our analysis are promising. Among the 46 HC-SMGs, eight, namely, *LYST* (lysosomal trafficking regulator), *DAPK1* (death-associated protein kinase 1), *CR2* (complement receptor type 2), *KIF16B* (kinesin family member 16B), *NPIPB15* (nuclear pore complex interacting protein family member B15), *SYTL2* (synaptotagmin-like protein 2), *ZNF91* (zinc finger protein 91), and *KIAA0586* (encodes the protein TALPID3, a centrosomal protein that is essential for primary cilia formation), which were mutated in 14.9% (152/1015) of CRC patients, have not been reported in any of the above databases. We speculate that these genes may be involved in the tumorigenesis or progression of CRC. The overall mutational landscape of HC-SMGs and the copy-number alterations are shown in Fig. 3c. A total of 83.6% (849/1015) of patients had at least one mutation within these HC-SMGs, with an average of 4.49 mutations in HC-SMGs per patient. We also estimated the frequency of loss of heterozygosity (LOH) for each HC-SMGs. As expected, *APC* and *TP53* are the top two genes that have the highest frequency of mutation combined with LOH (Supplementary Data 4).

We further compared the mutational frequencies of the HC-SMGs in both our dataset and three other large cohorts, namely, the TCGA, MSKCC, and DIFC, which mainly consist of Western populations (Supplementary Fig. 5). Although the total mutational frequencies of HC-SMGs were lower in our cohort than in the Western cohorts, the gene orders ranked by frequencies were comparative across each dataset (Supplementary Fig. 5), suggesting a similar importance of HC-SMGs between our cohort and the Western cohorts.

Mutational hotspots were also explored in this study. By utilizing a method described by Chang et al.^20^, we identified 52 substitution hotspots in 14 genes (Supplementary Data 5). Notably, a large fraction of these hotspots were not identified from the TCGA dataset. Taking the *APC* gene as an example, in the current study, 13 hotspots were identified, eight of which were not included in the hotspot list from the database. These hotspots were evenly distributed at the APC protein, and most of them had even higher mutational frequencies than the existing ones (e.g., p.R876 in Fig. 3d).

The primary tumor location in CRC has prognostic value, especially in advanced stages. Here, we further explored the associations between the primary tumor sites (including ascending, transverse, descending, and rectal) and genomic profiles. The clinical characteristics according to each site are presented in Supplementary Table 1, showing significant differences in age, TMB, pathological grade, and MSI status (chi-square test, *P* < 0.05). Other features, such as gender, smoking history, family history, and pathological stage, were evenly distributed in each group. We found that the OS outcome of each group had no distinct difference, with slightly different OS outcomes among the four groups in stage III (Supplementary Fig. 6). With HC-SMGs that had adequate mutational frequencies for comparison, we found that the mutational frequencies of 34 of the 46 HC-SMGs were significantly different (*P* < 0.05, Supplementary Fig. 7a). Most genes had a higher mutation frequency in ascending or transverse tumors than in descending or rectal tumors. However, for the ascending/transverse and descending/rectum comparison, there were significantly different mutational frequencies in genes such as *APC*, *TP53*, and *PIK3CA*, suggesting a necessity for a more detailed classification of primary tumor sites in CRC. For mutation type comparisons among primary tumor sites, we also observed obvious differences (Supplementary Fig. 7b). For example, although at low frequencies, we found that the *PTEN* gene was deleted, with a gradually increasing frequency from ascending tumors to the rectum, while both the deletion of *SMAD4* and amplification of *ERBB4* were enriched in descending or rectal tumors (*P* < 0.05). For mutational distribution comparisons, as expected, genes harboring mutations such as *APC* and *TP53* had a different mutation preference among each primer site, which may cause distinct protein functions in tumor cells (Supplementary Fig. 7c and Supplementary Fig. 8). Notably, *APC* p.R1450 was enriched in ascending tumors, while p.T1556 was enriched in transverse tumors, R876 was enriched in descending tumors, and p.R216 was enriched in rectal tumors, suggesting a mutational preference of *APC* in different sites.

### Somatic copy number variations and the mutational signature

Furthermore, we applied GISTIC2.0 to identify recurrent SCNV events and to assess the CNV burden in the cohort. Our results confirmed the previously well-defined recurrent somatic copy-number alterations (SCNAs) in CRC, including gains at chromosomes 1q, 7, 8q, 13q, and 20q and losses at 1p, 4, 5q, 8p, 14q, 15q, 17p, and 18p (Supplementary Fig. 9). In addition, we identified several other recurrent alterations in focal regions, including the gain at 10q and loss at 20p. The 10q amplification was associated with lung cancer progression and metastasis^21^, and the 20p deletion was also observed in myeloid malignancies^22^.

To explore the etiology, we performed mutational signature analysis using the proportions of the 96 possible trinucleotides by nucleotide context^23^. Instead of extracting new signatures from the mutation matrix, we mainly focus on the precise contribution of the existing mutational signatures, the etiology of which was already dissected. Therefore, A LASSO-based signature analysis approach was adopted as it could robustly determine active signatures with few mutations from whole-exome sequencing^24^. With the threshold of being present in more than 1% of CRC samples, we ultimately obtained more than 13 different COSMIC signatures that may contribute to CRC mutagenesis (Supplementary Fig. 10). Consistent with previous studies, the age-related signature (Signature 1) was the major etiology of mutagenesis in CRC, presenting in more than 84.2% of patients. Signature 3 (associated with failure of DNA double-strand break repair by homologous recombination), Signature 6 (related to defective DNA mismatch repair), and Signature 10 (related to POLE-induced ultrahypermutation) were present in 13.4%, 11.5%, and 5.9% of patients, respectively. Notably, although at low frequencies, we found that other signatures, such as Signature 9 (activity of AID during somatic hypermutation), Signature 8 (weak strand bias for C > A substitutions and unknown etiology), and Signature 16 (an extremely strong transcriptional strand bias for T > C mutations in the ApTpN context), were present in a certain fraction of patients (5.7%, 4.9%, and 2.6%, respectively), suggesting that other etiologies are responsible for the mutagenesis of CRC.

### Oncogenic alterations in the cell cycle and TGF-beta pathways are correlated with the dismal survival of CRC patients

**Fig. 4:**
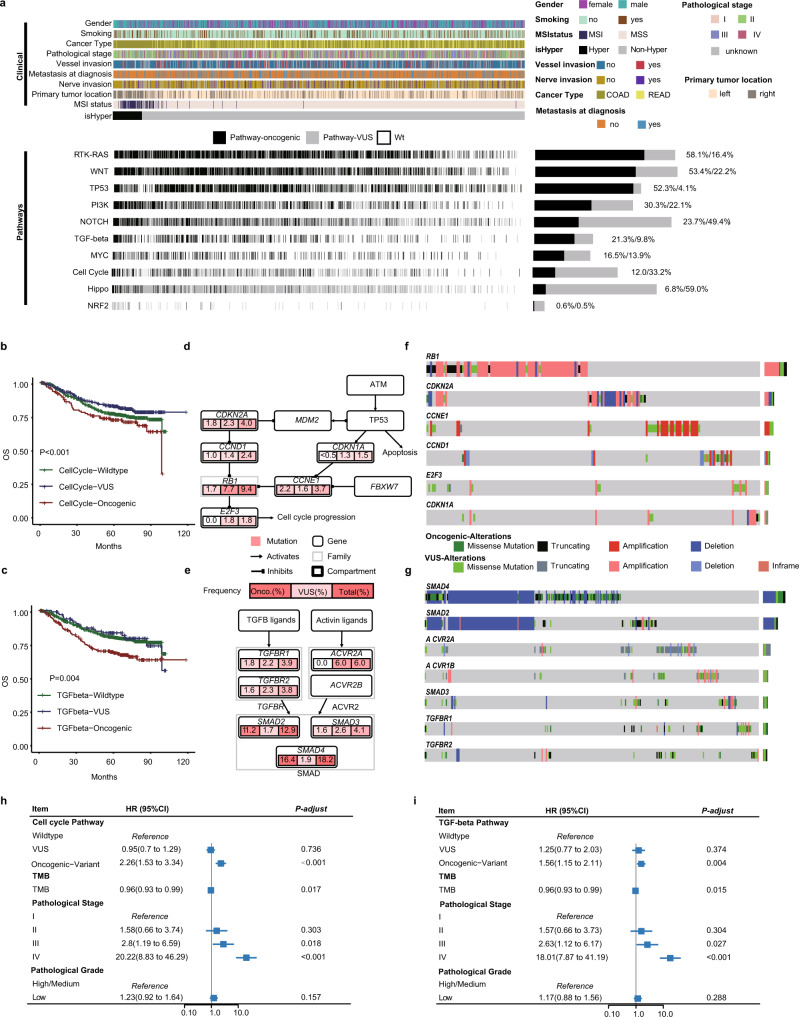
Alterations in oncogenic pathways in Chinese CRC patients. **a** Variant status of oncogenic signaling pathways in Chinese CRC patients. **Top:** Gender, smoking status, cancer type, pathological stage, vessel invasion, nerve invasion, metastasis at diagnosis, primary tumor location, MSI status, and hypermutated status. **Bottom right:** Variant status of 10 oncogenic pathways among the CRC patients (oncogenic variants denoted by black, variants of unknown significance (VUS) denoted by gray, wild type (Wt) denoted by white). **Bottom left:** Variant frequency of each oncogenic pathway. Kaplan–Meier estimates of OS in the Chinese cohort comparing patients carrying oncogenic variants, variants of unknown significance, and no variant (wild type) in the cell cycle pathway (**b**) or the TGF-beta pathway (**c**). Core members and interactions in the cell cycle pathway (**d**) and TGF-beta pathway (**e**). Genes are altered at different frequencies by oncogenic activation and tumor suppressor inactivation. Oncoplot for core members in the cell cycle (**f**) and TGF-beta (**g**) pathways. Forrest plot showing multi-variable Cox regression analysis of the effect of alterations in the cell cycle pathway (**h**) or the TGF-beta pathway (**i**) on CRC patients after adjusting for TMB, pathological stage, and pathological grade.

Next, we annotated mutated genes with cancer hallmark pathways to investigate the altered pathways and their associations with clinical factors. The landscape of genomic altered pathways and their clinical information are presented in Fig. 4a. To eliminate confounding impact from passenger mutations of tumor suppressor genes and oncogene, we categorized mutations or CNV events into either oncogenic variants or variants of unknown significance (VUS) based on the OncoKB database^18^. With the strategy, we found that large fraction of the alterations of several well-known signaling pathways including NOTCH, HIPPO, and the cell cycle were variants of unknown significance. After filtering for oncogenic events, correlation analysis between pathways and clinical factors was performed, and we found that except for nerve invasion and gender, all other clinical features correlated with at least one altered pathway among all cancer hallmark pathways (Supplementary Fig. 11a). Enrichment reanalysis further showed that the PI3K, cell cycle, and RTK-RAS pathways were significantly mutated in our cohort (all adjusted *P* values < 0.05).

Survival analyses (Supplementary Data 6) showed that patients with oncogenic alterations in the cell cycle and TGF-beta pathway had shorter overall survival than the respectively wildtype patients (P value < 0.001 and 0.004, respectively, Fig. 4b, c), which was consistent with previous reports^25,26^. In these two pathways, the *RB1*, *E2F3* and *ACVR2A* genes had relatively high fractions of VUSs (Fig. 4d, e). Specifically, we observed that amplification of the tumor suppressor gene *RB1* was classified as a VUS, indicating that the amplification of *RB1* is mainly a “passenger” event (Fig. 4d). *SMAD2* and *SMAD4* were the two top mutated genes in the TGF-beta pathway, and only oncogenic events were considered. The two genes had a high cooccurred deletion rate (18.2% of CRC patients) in the cohort (Fig. 4f and Supplementary Fig. 11b). However, for the cell cycle pathway, most of the altered genes, such as *RB1*, *CCND1*, and *CCNE1*, were mutually exclusively mutated (Fig. 4g and Supplementary Fig. 11c). Multiple regression analysis further demonstrated that the two pathways were independent factors for predicting patient outcomes after adjusting for known clinical risk factors, including TMB, pathological stage and ﻿pathological grade (﻿for the cell cycle pathway: HR = 2.26 (1.53–3.34), *P* < 0.001; for the TGF-beta pathway: HR = 1.56 (1.15–2.11), *P* = 0.004; Fig. 4h, i). We also investigated whether the combination of alterations in particular pathways confer poor prognosis (Supplementary Data 7). Interestingly, patients with mutations from both MYC and PI3K pathways had significantly worse overall survival than those with mutations in only one pathway or no mutations, even though mutations in only one pathway did not associate with a worse prognosis.

### Build genomic subtypes from genomic alterations of 1015 CRCs

**Fig. 5:**
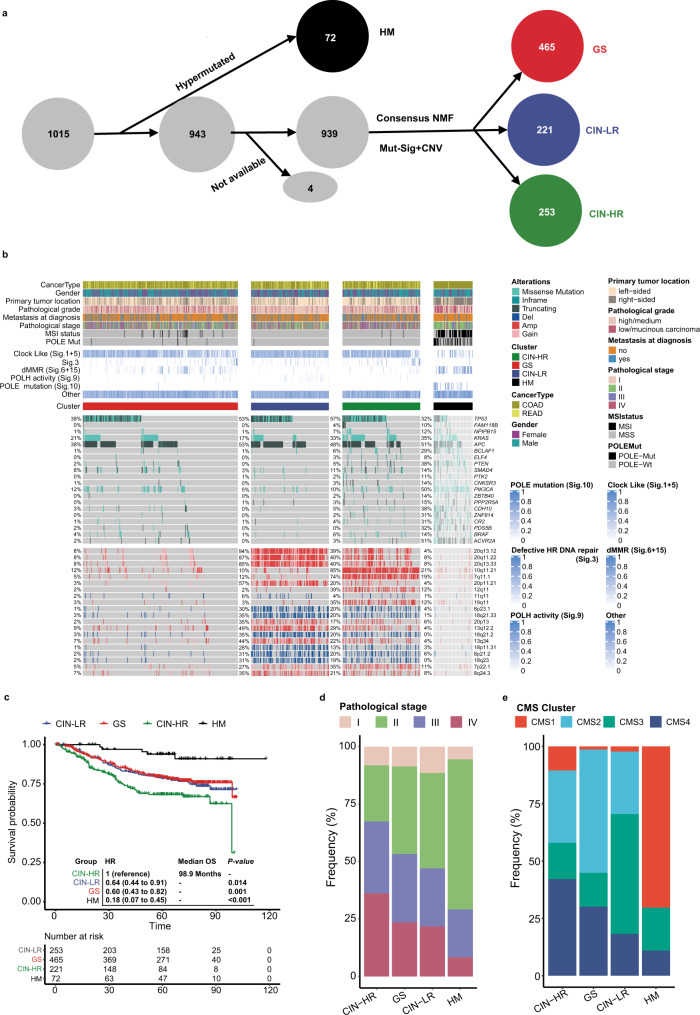
SYSUCC-CRC subtypes from 1015 Chinese patients with colorectal cancer. **a** Schematic diagram for the SYSUCC-CRC subtyping from genomic profile of CRCs. Tumor samples carrying hypermutation were first grouped as hypermutated (HM, black), and the remaining samples were grouped into three clusters using consensus NMF clustering: genomic stable (GS), chromosomal instability – low risk (CIN-LR), and chromosomal instability – high risk (CIN-HR) (see the main text, Methods and Supplementary Fig. 12). Four samples with no SCNV or mutational signature available were removed from the analysis. **b** Clinical features and molecular characteristics of the four subtypes. **Top**: Cancer type, gender, pathological stage, pathological grade, primary tumor location, metastasis at diagnosis, MSI status, *POLE* mutation status, mutational signature and subtype. **Middle**: The top 20 genes that were significantly differentially mutated in the nonhypermutated subtypes are ranked by the q value. Mutation color indicates the class of mutation. The percentage on the right side denotes the mutated frequency of each gene in each subtype. **Bottom**: The top 20 significant CNV lesions that were differentially altered in the nonhypermutated subtypes are ranked by the q value. Alteration color indicates the class of CNV. The percentage on the right side denotes the altered frequency of each lesion in each subtype. **c** Kaplan–Meier estimates of overall survival (OS) comparing the four CRC subtypes. **d** Distribution of pathological stage in the four subtypes. **e** Distribution of CMS subtypes across the four molecular subtypes in the TCGA-CRC cohort.

Based on all the somatic single-nucleotide variants (SSNVs) and SCNVs identified in our cohort, we sought to establish improved molecular subtypes of CRC (namely SYSUCC-subtypes). We first defined the hypermutated group as cluster 1 to avoid clustering bias from a high TMB. Then, we applied a nonnegative matrix factorization method (Supplementary Fig. 12) to classify the nonhypermutated patients into three clusters (Fig. 5a). Overall, the 1015 CRC patients were categorized into four subgroups, each with different genomic features and clinical characteristics. The four subgroups were defined as follows: (1) C1: hypermutated group with MSI-H or *POLE* mutations(HM); (2) C2: chromosomal instability with high risk (CIN-HR); C3: chromosomal instability with low risk (CIN-LR); and (4) genomic stable (GS) with relatively few somatic alterations and SCNVs (Fig. 5b). Then, we analyzed the associations between the different subgroups and clinical factors. As a result, the four subtypes exhibited significantly different overall survival outcomes (Fig. 5c and Supplementary Fig. 13a). Patients with the HM phenotype had the longest median overall survival, in line with previous clinical studies^10,12,27^. On the contrary, CIN-HR patients had a poorest overall survival compared to the other subtypes. Same tendency was observed in disease-free survival (Supplementary Fig. 13b, c). The CIN-HR subtype had a significant higher fraction of copy-number amplifications at 10q11.21 than CIN-LR, which could be used as biomarkers for distinguishing CIN-HR and CIN-LR (Supplementary Fig. 14a) and predicting CRC prognosis (Supplementary Fig. 14b, c). The fraction of the amplification was also significant higher in CRC patients with metastasis (Supplementary Fig. 14d) and increasing tendency was also detect in terms of the proportion of 10q11.21 amplification from stage II CRCs to stage IV CRCs (Supplementary Fig. 14e). Of note, the occurrence rate of 10q11.21 amplification was significantly lower in TCGA cohort (Supplementary Fig. 14f), probably due to the difference in metastasis proportion and racial composition. Though, CRC patients with 10q11.21 amplification also tend to have shorter OS and PFS than those without the amplification in the TCGA cohort (Supplementary Fig. 14g, h). 10q11.21 contains oncogenes such as *RET* and *NOTCH4*, which are associated with dismal survival in patients with CRC^28,29^. For the CIN-LR and GS subtypes, although no survival difference was observed, the differences in genomics suggest diverse mechanisms of tumorigenesis. Clinical association analysis showed that the fraction of stage IV tumors increased sequentially in the HM, CIN-LR, GS and CIN-HR subgroups (Fig. 5d), confirming the clinical relevance of these subtypes. Moreover, with pre-defined subtypes^30^ and copy-number amplification of 10q11.21, we also classified CRC patients from the TCGA cohort into CIN-LR, GS, CIN-HR and HM subgroups. By integrating the transcriptomic data and consensus molecular subtypes (CMS), we found that CIN-HR subgroups contained the largest fraction of CMS4 patients (Fig. 5e), which indicated the correlation between CIN-HR and activated stroma and might partially explain the prognosis value of CIN-HR and 10q11.21.

### Immunogenicity assessment and its clinical relevance in Chinese CRC Patients

**Fig. 6:**
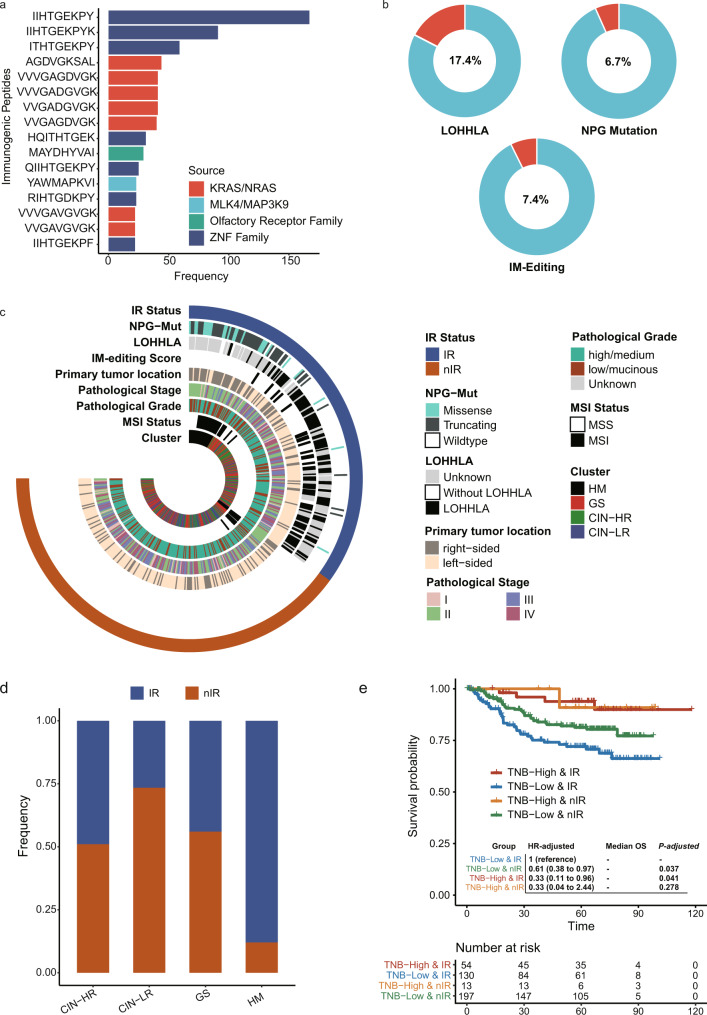
Immunogenicity reducing was associated with poor prognosis in CRC patients carrying low neo-antigen burden. **a** Top 16 variant peptides with highest frequency in CRC patients from SYSUCC cohort. **b** Frequency of different ways for neo-antigen presentation deficiency in CRC patients (LOHHLA: loss of heterogeneity in human leukocyte antigen; NPG: neo-antigen presenting genes; IM-editing status: immunoediting status). **c** Heatmap depicting the relationship across the status of immunogenicity-reduced status, clinical features, and molecular cluster (IR: immunogenicity-reduced; nIR: non-immunogenicity-reduced). **d** The difference on the fraction of IR and nIR comparing different molecular subtypes. **e** Comparison on overall survival across CRC patients classified by neo-antigen burden and status of immunogenicity reduction.

Immunogenicity of cancer mainly source from virus integration and variant peptides. The cancer-related virus sequences were examined specifically from the whole-exome sequencing dataset. EBV nucleic acid was detected in the tumor samples of 6% (61/1015) of patients, while HBV was detected in 2% (23/1015) of patients, and only one case had HPV infection. Potential neo-antigens were also predicted for each sample. With the density distribution analysis, we found that tumor neo-antigen burden(TNB) of most Chinese CRC patients was between 10 and 1000 (Supplementary Fig. 15a). The most frequent immunogenic peptides derived from the variation of ZNF family. Besides, variant peptides generated from mutations of *KRAS*/*NRAS* were among the top 10 most frequent neo-antigens (Fig. 6a).

Reducing immunogenicity by alteration of antigen presenting genes (NPG) and immunoediting of neoantigen is one of the most significant ways for tumor escaping from immune clearance. Alteration of NPG included mutation at NPG and the loss of heterogeneity in human leukocyte antigen (LOHHLA). Patients with LOHHLA account for 17.4% in 415 assessable CRC patients and 6.7% of 1015 CRC patients carried mutations at NPG (Fig. 6b and Supplementary Fig. 15b). Immunoediting of neoantigen could also be quantified by immunoediting score with information of mutations and neo-antigens^31^. As expected, immunoediting score in CRC population coincided with normal distribution (Supplementary Fig. 15c) and after Z-score conversion, we defined CRC patients with extremely low immunoediting score as immunoedited group and the remains as non-immunoedited group (Fig. 6b and Supplementary Fig. 15c). With mutation of NPG, LOHHLA and immunoedited status, we classified CRCs into immunogenicity-reduced (IR) group and non-immunogenicity-reduced (nIR) group (Fig. 6b, c, Methods). Additionally, HM subgroup had largest fraction of IR patients, which is followed by CIN-HR (Fig. 6d). We further classified CRC patients into four groups according to TNB and IR status. Similar to TMB, patients with high TNB had longer OS than those carrying low TNB (Fig. 6e). After correcting pathological stage and pathological grade, we found that patients with low TNB and IR had the worst overall survival, suggesting a correlation between the immunogenicity status and prognosis in CRC (Fig. 6e).

### Mitochondrial genomic alterations define a high-risk subgroup of Chinese CRC Patients

In addition to the coding regions of all human genes, WESplus also captures the whole genome sequence of mitochondria (MT). A recent study comprehensively characterized the MT genome from sequencing dataset in multiple cancers and revealed that alterations in the MT genome correlate with clinical characteristics and contribute to carcinogenesis by inducing abnormal energy metabolism^32^. In our study, we sought to investigate the MT genome in CRC and its clinical significance with improved probes and sample size.

SSNVs, including noncoding mutations, missense mutations, and other truncating mutations, were detected separately from the SSNVs in the nuclear genome. In total, we identified 2310 SSNVs in all samples, was accounting for 66.6% (676/1015) of patients with MT SSNVs (Supplementary Data 8). Among the mutations, genes that encode ribonucleotide-diphosphate reductase subunit 1/2 (RNR1 and RNR2) and D-loop were the three noncoding RNAs with the highest mutation rates (Supplementary Fig. 16a). Notably, as the genomic loci of *RNR1*, *RNR2*, and *D*-*LOOP* are physically adjacent, we speculated that the region containing these three genes was a mutation hotspot in the MT genome of CRC cells. Of the 13 protein-coding genes, *ND5*, *CYB*, and *ND4* were the top three most frequently mutated genes in CRCs, each of which was mutated in more than 10% of samples.

**Fig. 7:**
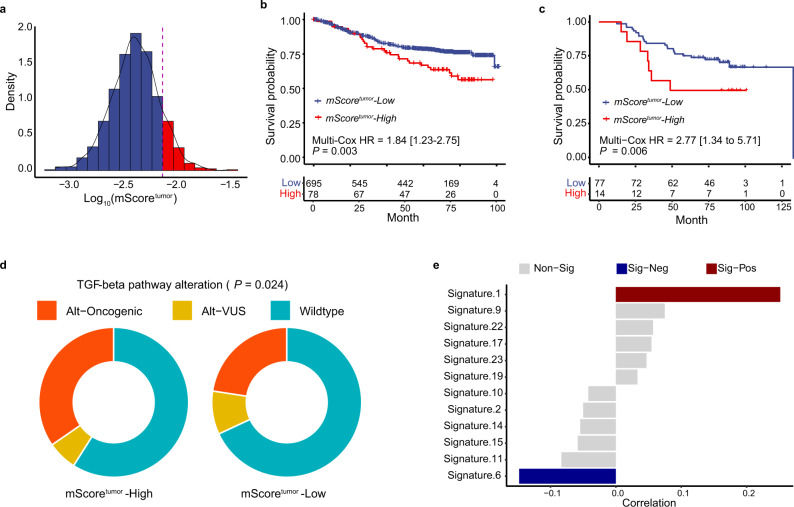
Characteristics of the mitochondrial genome in Chinese CRC patients. **a** Density distribution of the tumor mScore in all CRC samples, with the bottom 90% denoted by blue and the top 10% denoted by red. **b** Kaplan–Meier estimates of overall survival (OS) comparing CRC patients with a high tumor mScore (top 10%) and those with a low tumor mScore (bottom 90%). **c** Kaplan–Meier estimates of overall survival (OS) comparing CRC patients with a high tumor mScore and those with a low tumor mScore in qPCR-validating cohort. **d** Difference on the alterative frequency of TGF-beta pathway comparing patients with a high mScore^Tumor^ and those with a low mScore^Tumor^. **e** Correlation between mutation signature and mScore^Tumor^ (Non-Sig: non significantly; Sig-Neg: significantly negative correlation; Sig-Pos: significantly positive correlation).

MT genomic copies were assessed and normalized by the purity of each sample. Here, we defined the normalized MT copies as the mScore. In line with previous reports, MT copies represented by the mScore from tumor tissues were significantly lower than those from normal adjacent tissues (*P* < 0.001, Supplementary Fig. 16b) and mScores from tumor tissues were positively correlated with those from the paired normal tissues (*P* < 0.001, Supplementary Fig. 16c). We then divided the whole cohort into the mScore-high and mScore-low subgroups by utilizing the best-cutoff strategy (Fig. 7a). We found that top 10% of CRC patients with extremely high MT copies (mScore-high) had significantly worse overall survival than those with a low mScore (mScore-low) (Fig. 7b), which was independent from the known clinical factors, including pathological stage and pathological grade (HR = 1.84, 1.23–2.75, *P* = 0.003, Supplementary Fig. 16d). To validate the prognostic value of mScore and simplified the usage in clinical practices, we tested if qPCR could accurately quantify the mScore of CRC samples as the WES did. As expected, relative mScore of tumor quantified by qPCR (mScore-qPCR) was significantly correlated with mScore estimated from the WES (mScore-WES) (Supplementary Fig. 16e). Moreover, we defined the optimal cutoff of mScore-qPCR with liner regression and preset cutoff of mScore-WES and classified patients from an independent CRC cohort as mScore-high group and mScore-low group. Similarly, the mScore quantified by qPCR could still predict the prognosis of CRC patients in the independent cohort after correcting pathological stage and pathological grade (Fig. 7c). The results suggested that the mScore could serve as a promising prognostic biomarker for CRC.

To further characterize the clinical relevance of the mScore, we performed association analyses between the mScore and the known clinical factors. Most of the clinical risk factors, including pathological stage, pathological grade, nerve invasion, and vessel invasion, were not correlated with the mScore (Supplementary Fig. 17).

By integrating the alteration on chromosome, we found that CRC patients with high mScore carried a higher alteration frequency of TGF-beta pathway (Fig. 7d), higher mutation frequency of *TP53* and lower frequency of *DICER1* (Supplementary Data 9). Besides, mScore was positively correlated with mutation burden deriving from signature 1 (clock-like signature) while negatively correlated with mutation burden deriving from signature 6 (dMMR signature)(Fig. 7e).

## Discussion

In this study, we sequenced the DNAs of more than one thousand CRC patients with ultradeep whole-exome sequencing, generating a large CRC cohort with relatively complete genomic and clinical information of CRC. With improved sequencing depth, coverage, and sample size, our study provides improved statistical power to the identification of genomic alterations with low frequencies and refine the association between the known genomic variables and clinical features. In addition, with a rich clinical annotation of family cancer history, treatment information, disease-specific outcome and long term follow-up, our dataset provides a landmark resource for exploring the genetic risks related to CRC and identifying extra biomarkers to predict prognosis and the response/resistance to therapy. Recently, immunotherapy represented by immune checkpoint inhibitors have become a major area of interest in the treatment of colorectal cancer^33^ and the FDA approved the pembrolizumab (an anti-PD-1 monoclonal antibody) for adults and children with TMB-high solid tumors^34^. However, such approval is based on the tests of gene panels with limited gene numbers. TMB derived from whole-exome sequencing is considered the gold standard in clinical practice. Therefore, an accurate estimation of each patient’s TMB would be of great value for identifying those appropriate for CRC treatment. Here, our study provided thousand WES-based TMB baseline data for better defining TMB-high and TMB-low in terms of immunotherapy in Asian CRCs.

The current study utilized an integrated approach to identify HC-SMGs from the whole exome and to establish an alternative genomic-based subtyping system for CRC. For cancer genomic studies, defining driver genes by a single algorithm usually introduces false-positive records. We proposed that the potential driver genes identified by multiple tools can produce high priority scores for further investigation and that the discovery of new SMGs could provide new underlying mechanisms of CRC tumorigenesis. Here we selected five tools mainly referenced from a recent published esophageal adenocarcinoma genomic study^35^, the core principle of which were sought to represent the most known tools. Therefore, some other SMG identification algorithms such MutPanning^36^ and RF5^37^ were not included in the result. As those tools also had great performances for the purpose, we expected an integrative analysis of SMGs in the future that would include more tools and larger samples with the help of our dataset. In the current study, we obtained 46 HC-SMGs, eight of which were mutated in approximately 15% of patients in our cohort and 19.55% in the TCGA cohort. Among those genes, only *DAPK1* and *SYTL2* have been implicated in cancers. Specifically, *DAPK1* encodes a structurally unique 160-kD calmodulin-dependent serine-threonine kinase that is involved in multiple cellular signaling pathways, including cell survival, apoptosis, autophagy^38^, and both type I and type II autophagic cell death signals. *DAPK1* was reported to be associated with only pancreatic ductal adenocarcinoma^39^. *SYTL2* encodes a synaptotagmin-like protein (SLP) that belongs to a C2 domain-containing protein family^40^. The SLP homology domain (SHD) of this protein can bind specifically to the GTP-bound form of Ras-related protein Rab-27A (RAB27A)^41,42^. Diseases associated with *SYTL2* include Griscelli syndrome and type 1 and type 2 Griscelli syndrome^43^. Its related pathways are Rab and Rab effector genes in bladder cancer^44^. Notably, the eight HC-SMGs identified in this study have not been included in current known gene panels (known panel information from the cBioPortal database, summarized in Supplementary Data 10), suggesting a need for whole-exome sequencing or the extension of current gene panels in the clinical genetic testing of CRC.

With the somatic mutations and copy-number variations estimated from WES, we reconstructed the current CRC genomic subtypes (like GS and HM) and provide an improved genomic subtyping system based on unsupervised approaches and clinical features. Combined with the hypermutated phenotypes of CRC, we divided the whole CRC cohort into four subtypes with distinct survival endpoints. Regarding clinical usage, compared to the molecular subtypes derived from a transcriptomic dataset^5^, genomic-based clustering is simpler to use and more feasible. Among the four subtypes, HM and GS are two well-known features of CRC, reflecting the two extreme conditions of CRC genomics. For CIN-HR and CIN-LR, we associated the molecular subtypes with clinical outcomes. Compared with HM and GS, these two subtypes have intermediate genomic alterations but are characterized by different clinical phenotypes, indicating that these two subtypes are driven by distinct molecular mechanisms.

Another feature of this study is that we performed absolute quantification and variant detection at the mitochondrial genome level in CRC tumor and normal samples. Previous pan-cancer studies comprehensively analyzed the mitochondrial genomic variants with whole-genome sequencing data and found high mutational defects in many cancer types^32^. In our study, we confirmed these findings with an alternative method and then identified a subgroup of CRC with an active oxidative phosphorylation phenotype represented by high mitochondrial copy numbers. Notably, a recent study by Sun et al. proved in an animal model that an increased mtDNA copy number promotes microsatellite-stable (MSS) CRC progression by enhancing oxidative phosphorylation^45^. This provides a potential mechanism for the findings. Moreover, we proposed an optimized mScore(representing the mtDNA abundance) cutoff to divide patients into high and low risk group, which was further validated in an independent cohort using the qRT-PCR technology. The later was a well-developed clinical feasible detecting method and thus could simplified the mScore estimation in CRC patients, suggesting a further usage of the mScore for CRC patients’ stratification.

Our study provides a high-quality genomic resource and adds to the existing datasets by characterizing the genomic landscape of a large cohort of Chinese CRC patients. To facilitate board usage of the data, we built an online resource for easy access to a processed analysis-ready dataset (https://changkang.hapyun.com/). In summary, combined with the HC-SMG genes, high mitochondrial copy number subgroup, associated pathways and refined genomic subtypes of CRC, our study successfully identified rich connections between genomic variations and clinical characteristics. We believe that our dataset and the analyses presented in this study will be broadly used to discover new biological mechanisms and therapeutics for this deadly disease.

## Methods

### Sample enrollment

The study protocol was approved by Sun Yat-sen University Cancer Center Ethics Committee (B2019-031-01, Guangdong, China) and written informed consent was obtained from all patients. No statistical methods were used to predetermine sample size. For sample enrollment, the patients that were diagnosed as colorectal cancer with histological confirmed to be adenocarcinoma were enrolled, following the inclusion criteria: (1) between 18 and 80 years old; (2) received resection of the primary tumor in Sun-Yat Sen University Cancer Center; (3) enough tumor sample for next-generation sequencing; (4) no prior chemotherapy, radiotherapy, immunotherapy or other anti-tumor treatment at sampling point; (5) no history of other malignancy. Each sample was confirmed to contain at least 30% of tumor cells by two pathologists.

### DNA extraction and whole-exome sequencing

Genomic DNA from formalin-fixed paraffin-embedded (FFPE) colorectal tumors and patient-matched NATs (normal adjacent tissues) were extracted using the QIAamp DNA FFPE tissue kit (Qiagen). Extracted DNA was then quantified by Qubit 3.0 (Thermo Fisher Scientific, Inc., Waltham, MA, USA), in accordance with manufacturer’s instructions. DNA was sheared using enzyme dsDNA Fragmentase (New England BioLabs, Inc., Ipswich, MA, USA). Size selection of the DNA fragments (150–250 bp) was then performed using Ampure XP beads (Beckman Coulter, Inc., Brea, CA, USA), which has the additional benefits of higher recovery and greater speed. DNA fragments were used for library construction using the KAPA Library Preparation kit (Kapa Biosystems, Inc., Wilmington, MA, USA) according to the manufacturer’s protocol. Agencourt AMPure XP beads (Beckman Coulter, Inc., Brea, CA, USA) were used for all the cleanup steps. End repair and 3′-end A-tailing were performed following DNA fragmentation. The purity and concentration of the DNA fragments were assessed using the Qubit 3.0 fluorometer and the Qubit dsDNA HS Assay kit. Exonic regions of DNA were enriched with WESPlus gene panel which is an upgraded version of the standard whole-exome sequencing (HaploX Biotechnology, Shenzhen, China), and 150 bp paired-end sequencing was performed on NovaSeq 6000 system (Illumina).

### Raw data processing

Raw sequencing reads were preprocessed by fastp v0.12.6^46^ for subsequent analysis: (1) adapter trimming; (2) remove the reads in which the N base has reached a certain percentage (default length of 5 bp); (3) remove the reads which contain low-quality bases (default quality threshold value < = 20) above a certain portion (default 40%); (4) sliding window trimming: the bases in the sliding window (default is 4 bp) with mean quality below cutting quality (default is 20) will be cut.

### Alignment and somatic mutation calling for genomes

The cleaned reads were aligned to the reference human genome (build hg19) using Sentieon bwa-mem. Subsequent processing including sorting reads and marking duplicates were performed using Sentieon tools v201808 (https://www.sentieon.com/), which were improved upon the GATK Toolkit (https://gatk.broadinstitute.org/hc/en-us). Sequence depth and coverage were obtained using bamdst (https://github.com/shiquan/bamdst). To identify all the variants, we used three somatic mutation callers for single nucleotide variants (SNVs) and indels: VarScan2 (v2.3.8) (-min-coverage 10 -min-var-freq 0.05 -min-freq-for-hom 0.75 -*p*-value 0.99 -somatic-*p*-value 0.05)^47,48^, Mutect2 (gatk mutect2 -R -pon -germline-resource -af-of-alleles-not-in-resource 0.0000025 -L -disable-read-filter)^49^ and TNscope (sentieon driver -t -algo TNscope -dbsnp -pon -cosmic). To improve specificity, a panel of normal^17^ sample filtration was used to remove background germline variations and artifacts. Mutect2 and TNscope were based on bam files which processed by quality score recalibration that performed using GATK4 (v 4.1.1.0)^50^. Specifically, processSomatic and somaticFilter (-min-coverage 10, -min-reads2 2, -min-strands2 1, -min-avg-qual 20, -p-value 0.05) were used to extract high-confidence somatic variants from the raw VarScan2 results and to remove clusters of false positives and SNVs near indels. Additional filtration with fpfilter.pl script from Varscan2 and tumor read counts generated by bam-readcount (https://github.com/genome/bam-readcount) was performed to reduce false-positive calls. We discarded SNVs in RepeatMasker repeat regions (http://www.repeatmasker.org). Somatic mutations were then annotated using VEP^51^. To obtain reliable mutation calls, we used a two-step approach. First, chose mutations that were identified in at least two of the three callers (Mutect2, TNscope, and VarScan2). Second, additional filtering with three criteria was performed: (1) variant allele frequency (VAF) > = 8%; (2) sequencing depth in the region > =8; (3) sequence reads in support of the variant call > = 2.

### TMB definition and calculation

Tumor mutation burden (TMB) was defined as the number of somatic mutations per Mb that (1) only consider the following functional classification: Frame_Shift_Del, Frame_Shift_Ins, In_Frame_Del, In_Frame_Ins, Missense_Mutation, Nonsense_Mutation, Nonstop_Mutation, Splice_Site; (2) removed the common variants in 1000 genomes (MAF > 0.05) and the exome aggregation consortium (EXAC, MAF > 0.05).

### HC-SMG identification

To detect recurrent mutated gene, a multi-tool approach was implemented to provide a comprehensive analysis. We used five computational tools: dNdScv^52^, e-driver^53,54^, MutSig2CV^55^, OncodriveCLUST^56^ and OncodriveFM^57^. Of the five computational tools, dNdScv, MuSig2CV and OncodriveCLUST are based on mutation frequency; OncodriveFM is based on functional impact; e-Driver is based on structural genomics. dNdScv, e-driver, MutSig2CV and OncodriveCLUST were run with the default parameters. Genes were deemed significant at a q-value of 0.1; The rational of each tool was described as follows: dNdScv is a suite of maximum-likelihood dN/dS methods. The background mutation rate of each gene is estimated by combining local information (synonymous mutations in the gene) and global information (variation in the mutation rate across genes, exploiting epigenomic covariates), and controlling for the sequence composition of the gene and mutational signatures. dNdScv uses trinucleotide context-dependent substitution matrices to avoid common mutation biases affecting dN/dS; MutSigCV incorporates features including point mutations, small insertions/deletions, coverage to identify SMGs. To reduce false discovery rate, it also corrects the variation from patient-specific mutation frequencies, mutation spectra, gene-specific mutation rates, expression levels and replication times. MutSigCV is especially useful for tumor samples with high mutation rates; OncodriveCLUSTL is featured by detecting significant clustering signals across genomic regions. It could detect genes with a significant bias toward mutation clustering in specific protein regions using silent mutations as a background mutation model. To note, OncodriveCLUSTL was run with parameters: a maximum cluster distance of 3, a minimum number of mutations for a gene of 7, and a probability of the binomial model to find cluster seeds of *P* = 1 × 10e−13 to exclude probable false-positive genes; OncodriveFM identify driver genes or gene modules by computing a metric of functional impact using three well-known methods (SIFT, PolyPhen2, and MutationAssessor) and then evaluating the functional impact of variants found in a gene across several tumor samples deviates from a null distribution; e-Driver identifies protein regions that are enriched in somatic missense mutations using a binomial test and assuming mutations are distributed randomly across the protein. Collectively, we used candidate genes identified in either method or merge them together.

### Somatic copy number analysis and recurrent SCNV identification

Somatic copy number variations were analyzed as previously descripted^58^. In brief, we applied EXCAVATOR2 v1.1.2^59^ to estimate the copy ratio information from WES dataset. EXCAVATOR2 is a collection of bash, R, and Fortran scripts and codes that analyses WES data to identify CNVs. It extends the Read Count approach to the whole genome sequence and exploits the Shifting Level Model (SLM) algorithm to segment the two combined profiles. The ABSOLUTE package v1.0.6^60^ was used to estimate the purity and the average ploidy for each tumor sample. We used both the segmented copy ratio results (the log_2_ copy number change) and the allelic depth of somatic mutations for the estimation of the purity. To study significantly recurrent regions of SCNA, we applied GISTIC2 v2.0.23^61^ applied to the copy number segments. GISTIC2 was run with the following parameters: -ta 0.3 -td 0.3 -armpeel 1 -cap 1.5 -conf 0.99 -genegistic 1 -gcm mean -js 4 -maxseg 2000 -qvt 0.05 -savegene 1 -brlen 0.98 -broad 0 -rx 0.

### Mutational signature and pathway analysis

Only variants with the following functional classification were considered in this part: missense mutation, nonsense mutation, nonstop mutation, RNA mutation, silent mutation, variants at splice site or translation start site, insertion and deletion. Mutational signatures were identified using R package LassoSig^62^ from the CRC samples (only samples with at least 20 SNVs were included). The normalization method was set to ‘exome2genome’. This approach organized sample information in the form of the fraction of mutations in each of 96 trinucleotides and determined the weighted combination of the COSMIC signatures (https://cancer.sanger.ac.uk/signatures/signatures_v2/)^23^ that most closely reconstructed the mutational profile.

For pathway analysis, functional enrichment analysis was carried out with Fisher’s exact test as implemented in the clusterProfiler^63^ Bioconductor package, with a Bonferroni correction and an adjusted *p*-value of 0.05. Cancer Hallmarks annotations were collected from GSEA database. For oncogenic alteration, genomic alterations were annotated for oncogenic variants using OncoKB Annotator (Chakravarty et al., 2017), a precision oncology knowledgebase that tracks the effects of cancer variants and their potential clinical actionability (http://oncokb.org).

### Mitochondrial genome somatic mutation calling and mtDNA copy number estimation

We used Samtools^64^ *bam2fq* function to extract the reads specifically mapped to mitochondrial genome from the whole genome mapped BAM file. Those reads including perfect matched reads and non-perfect matched reads for the further analysis that could be distinguished by blastn program. For mutation analysis, only non-perfected mapped reads were used. We applied SPAdes^65^, a de novo assembly tool, to assembly the sample specific whole MT genome with ‘--only-assembler mode’. For the assembly result, we only consider the longest contig that has more than 16k nt and adequate depth for mutation calling. By mapping the assembled configs to reference MT genome via minimap2^66^ (Parameter: -ax asm5 --cs=short --secondary no), we can sequentially obtain the SNP information from the mapped result. Next, the SNPs called from tumor samples and not exist in paired normal samples were considered as the somatic mutation candidates. Among those candidates, four sites were strictly removed as they may result in misalignment in genome, according to the previous study^67^: three positions encoded as “N” to preserve historical numbering, (523, 524, and 3107), in addition position 310 is located within a homopolymer region and is a common variant.

For mtDNA copy number quantification, we applied an R package ExomeDepth^68^ to estimate the raw copy number of each sample. To calculate the normalized copy number of mitochondrial genomes, we first calculated the purity/ploidy corrective factor ‘**R**’ (Eq. 1). Then, we applied ‘R’ to correct the ratio of (1) the number of sequencing reads mapping to the MT genome (rm) to (2) the number of reads mapping to the nuclear genome (rn) and subsequently obtained **m** value with Eq. 2. Assuming two samples have been processed in identical manners, the sample with a higher value of **m** contains more copies of mtDNA.

1 $${R}_{{Tumor}}=\frac{{Purity}\times {Ploidy}+(1-{Purity})\times 2}{2}$$

2 $$m=\frac{{r}_{m}}{{r}_{n}}\times R$$

As for qPCR method, we first extracted genomic DNA from tumor tissue using TIANamp Genomic DNA Kit (Cat#DP304-03, Tiangen). MtDNA genomic copies was determined by the relative expression level of beta-2-microglobulin (endogenous control) and a D-loop fragment contained in mitochondrial genome. 10 ng DNA was added as template for each reaction, and qPCR was completed using the GoTaq qPCR Master Mix (Cat#A6002, Promega) in LightCycler 480 instrument (Roche Diagnostics, Switzerland). Primers used in this study sourced from Osch et al.^69^ as follows: for beta-2-microglobulin, forward primers TGCTGTCTCCATGTTTGATGTATCT’, reverse primers ‘TCTCTGCTCCCCACCTCTAAGT’; for D-loop fragment, forward primers ‘CATCTGGTTCCTACTTCAGGG’, reverse primers ‘TGAGTGGTTAATAGGGTGATAGA’. The PCR reaction conducted at an initial 2 min at 50 °C, then 10 min at 95 °C, followed by 40 cycles of 15 s at 95 °C, and 1 min at 60 °C. Finally, MtDNA genomic copies were calculated using 2-ΔΔCt method.

### Non-negative matrix factorization consensus clustering

The consensus non-negative matrix factorization (CNMF) method was applied to the continuous variable matrix of mutation signature and the discrete variable matrix of lesion SCNAs with varying the number of clusters from *K* = 2 to 6. Four patients lacking sufficient information were excluded from the clustering. Based on the visual inspection of a hierarchical clustering of the consensus matrix and optimal cluster number, defining the average connectivity over 10 cluster runs with different initial condition, the case of *K* = 3 was used to arrange samples. The CNMF method was performed using the R package ‘NMF’ in R (http://cran.r-project.org/package=NMF). Taking the hypermutated patients together, we named the four subgroups according to their molecular characteristic and prognosis. By comparing the molecular characteristics between CIN-HR and CIN-LR, we found that amplification of 10q11.21 lesion could help distinguish the two subtypes. Accordingly, with copy number variation (CNV) data of colorectal cancers from TCGA database, we defined cancers that carried amplification of at least one gene in 10q11.21 as cancers with amplification of group. A previous study had classified CRC from TCGA database as GS, CIN, HM^30^. Accordingly, we further classified the CIN subgroup as CIN-HR patients and CIN-LR patients with the status of 10q11.21 amplification.

### Analysis of immune-associated features

HLA class I typing of SYSUCC-CRC samples was performed on WES data from normal tissues with POLYSOLVER^70^. With the HLA types, potential neoantigenic peptides were identified using NeoPredPipe^71^ with default arguments, based on the somatic nonsynonymous coding single nucleotide variants and somatic indel variants. Loss of heterogeneity in human leukocyte antigen (LOHHLA) was performed with LOHHLA tools (https://bitbucket.org/mcgranahanlab/lohhla/src/master/), based on the algorithm from McGranahan et al.^72^. Since some HLA alleles were not found in the database of HLA-I types used by LOHHLA, we only took into consideration the samples that had intact outputs of HLA-A, HLA-B and HLA-C. Accordingly, 415 CRC samples was remained for further LOHHLA assessment. With the standard described by Lakatos et al.^73^, a sample was considered to have allelic imbalance at an HLA locus if the corresponding P value was below 0.01 and LOH if, in addition, the copy number prediction of that allele was below 0.5, with the confidence interval strictly below 0.7. Immunoediting score was calculated according to a previous published method^31^. Specifically, using the whole CRC dataset, we first derived the average number of immunogenic mutations per non-silent mutation for each trinucleotide context ${\bar{B}}_{s}$, (only samples with ≥ 10 non silent mutations were taken into consideration). For each spectrum ${\bar{B}}_{s}$, the expected number of non-silent mutations per silent mutation, ${\bar{N}}_{s}$, was estimated. Given a set of silent mutations, ${S}_{i}$ and their corresponding mutation context *s(m)*, in the sample $i$, the expected number of nonsynonymous ${N}_{{pred},i}$and immunogenic mutations ${B}_{{pred},i}$ were calculated as:

3 $${N}_{{pred},i}=\mathop{\sum }\limits_{m}^{{s}_{i}}{\bar{N}}_{s\left(m\right)}$$

4 $${B}_{{pred},i}=\mathop{\sum }\limits_{m}^{{s}_{i}}{\bar{N}}_{s\left(m\right)}{\bar{B}}_{s\left(m\right)}$$

The immunoediting score represented the ratio of expected to observed immunogenic mutations per non-silent mutation.

5 $${I}_{i}=\frac{\frac{{B}_{{obs},i}}{{N}_{{obs},i}}}{\frac{{B}_{{pred},i}}{{N}_{{pred},i}}}$$

After Z-score conversion of ${I}_{i}$ we defined samples with converted immunoediting score below –1.645 were immunoedited. As mutations of antigen-presenting genes, LOHHLA and immunoediting of neoantigen are the three ways for tumors to reduce their immunogenicity and escape from immune clearance, we defined tumors with one of these characteristics as immunogenicity-reduced and those without all of these characteristics as non-immunogenicity-reduced.

### Statistical analysis

Statistical analysis was performed under R programming environment (https://www.r-project.org/) in version 4.0.1. Comparison between groups were examined either by Student’s *t*-test, nonparametric test or ANOVAR. Benjamini–Hochberg procedure was used to correct *P* values for multiple hypotheses testing when appropriate. Overall survival (OS) was defined from date of disease diagnosis to date of death or last available follow-up. Kaplan-Meier survival curves were generated and compared using the log-rank test. Multivariate analysis was performed with COX regression analysis. *P* < 0.05 were considered as statistically significant and labeled as *, while ** represents *P* < 0.01 (highly significant), and *** represents *P* < 0.001 (very highly significant), respectively.

### Reporting summary

Further information on research design is available in the Nature Research Reporting Summary linked to this article.

## Supplementary information

Supplementary Information

Supplementary Data

Reporting Summary

## Data Availability

Our study is compliant with the “Guidance of the Ministry of Science and Technology (MOST) of China for the Review and Approval of Human Genetic Resources”. The raw sequence data reported in this paper have been deposited in the Genome Sequence Archive in National Genomics Data Center, China National Center for Bioinformation / Beijing Institute of Genomics, Chinese Academy of Sciences, under accession number HRA000873 (Project: PRJCA004622). These data are available under restricted access, as patients were consented for sample collection which was stated for academic and non-profit purpose. The raw data are available for academic purpose and within the limitations of the provided informed consent, which can be granted by the Data Access Committee (DAC). Access can be obtained by completing the application form via GSA-Human System. For detailed guidance on making the data access request, see GSA-Human_Request_Guide_for_Users [https://ngdc.cncb.ac.cn/gsa-human/document/GSA-Human_Request_Guide_for_Users_us.pdf]. The approximate response time for accession requests is about 2 weeks. The processed, structured genomic dataset are available at the home page of the Changkang Project (https://changkang.hapyun.com/). Clinical data of the cohort are deposited as Supplementary Data 2.

## Code availability

The processed, structured genomic dataset and analysis code are available at the home page of the Changkang Project (https://changkang.hapyun.com/).
