# Supplementary material for: Comprehensive profiling of 1015 patients’ exomes reveals genomic-clinical associations in colorectal cancer
Source: Nat Commun. 2022 Apr 29;13:2342. doi: 10.1038/s41467-022-30062-8 (PMC9055073; doi:10.1038/s41467-022-30062-8)
Supplement: Supplementary file 1 — Supplementary Information [file 41467_2022_30062_MOESM1_ESM.pdf]

Supplementary Table 1

	Ascending (N=195)	Transverse (N=136)	Descending (N=359)	Rectum (N=316)	Unknown (N=9)	*P*-value
Age						
Mean (SD)	56.8 (12.9%)	55.6 (13.8%)	57.6 (12.5%)	59.0 (11.6%)	55.0 (12.7%)	0.070
Median [Min, Max]	56.00 [25.0, 83.0]	58.00 [20.0, 85.0]	59.00 [22.0, 85.0]	59.00 [18.0, 88.0]	56.00 [33.0, 73.0]	0.172
Sex						0.201
female	91 (46.7%)	51 (37.5%)	135 (37.6%)	122 (38.6%)	5 (55.6%)	
male	104 (53.3%)	85 (62.5%)	224 (62.4%)	194 (61.4%)	4 (44.4%)	
Smoking history						<0.001*
yes	42 (21.5%)	31 (22.8%)	77 (21.4%)	89 (28.2%)	1 (11.1%)	
no	153 (78.5%)	105 (77.2%)	281 (78.3%)	225 (71.2%)	7 (77.8%)	
Missing	0 (0.0%)	0 (0.0%)	1 (0.3%)	2 (0.6%)	1 (11.1%)	
Family history						0.236
yes	52 (26.7%)	33 (24.3%)	85 (23.7%)	58 (18.4%)	2 (22.2%)	
no	143 (73.3%)	103 (75.7%)	274 (76.3%)	258 (81.6%)	7 (77.8%)	
TMB						<0.001*
Mean (SD)	9.83 (23.42%)	9.12 (33.18%)	2.97 (6.90%)	1.97 (5.14%)	5.63 (9.24%)	
Median [Min, Max]	2.1 [0.1, 172.2]	2.1 [0.04, 339.7]	1.7 [0.01, 99.0]	1.5 [0, 90.9]	1.8 [0.2, 28.0]	
Stage						0.852
I/II	95 (48.7%)	67 (49.3%)	159 (44.3%)	149 (47.2%)	5 (55.6%)	
III/IV	100 (51.3%)	69 (50.7%)	200 (55.7%)	166 (52.5%)	4 (44.4%)	
Missing	0 (0%)	0 (0%)	0 (0%)	1 (0.3%)	0 (0%)	
Grade						<0.001*
High/medium	128 (65.6%)	92 (67.6%)	259 (72.1%)	252 (79.7%)	6 (66.7%)	
Low	65 (33.3%)	42 (30.9%)	95 (26.5%)	62 (19.6%)	3 (33.3%)	
Missing	2 (1.0%)	2 (1.5%)	5 (1.4%)	2 (0.6%)	0 (0%)	
Metastasis at diagnosis						0.253
yes	55 (28.2%)	33 (24.3%)	93 (25.9%)	71 (22.5%)	0 (0.0%)	
no	140 (71.8%)	103 (75.7%)	266 (74.1%)	245 (77.5%)	9 (100.0%)	
MSI status						<0.001*
MSI-H	35 (17.9%)	24 (17.6%)	16 (4.5%)	0 (0.0%)	1 (11.1%)	
non-MSI-H	160 (82.1%)	112 (82.4%)	343 (95.5%)	316 (100.0%)	8 (88.9%)	
MMR status						<0.001*
dMMR	33 (16.9%)	27 (19.9%)	18 (5.0%)	10 (3.2%)	0 (0.0%)	
pMMR	132 (67.7%)	90 (66.2%)	297 (82.7%)	259 (82.0%)	7 (77.8%)	
Missing	30 (15.4%)	19 (14.0%)	44 (12.3%)	47 (14.9%)	2 (22.2%)	

Supplementary Table 1. Clinicopathologic Features among different colorectal cancer locations. For continuous variable parametric test, ANOVA test were used. For categorical variable test, one of either “Chisq” or “Fisher” were used. *P < 0.05.

Supplementary Figures

Supplementary Figure 1

a

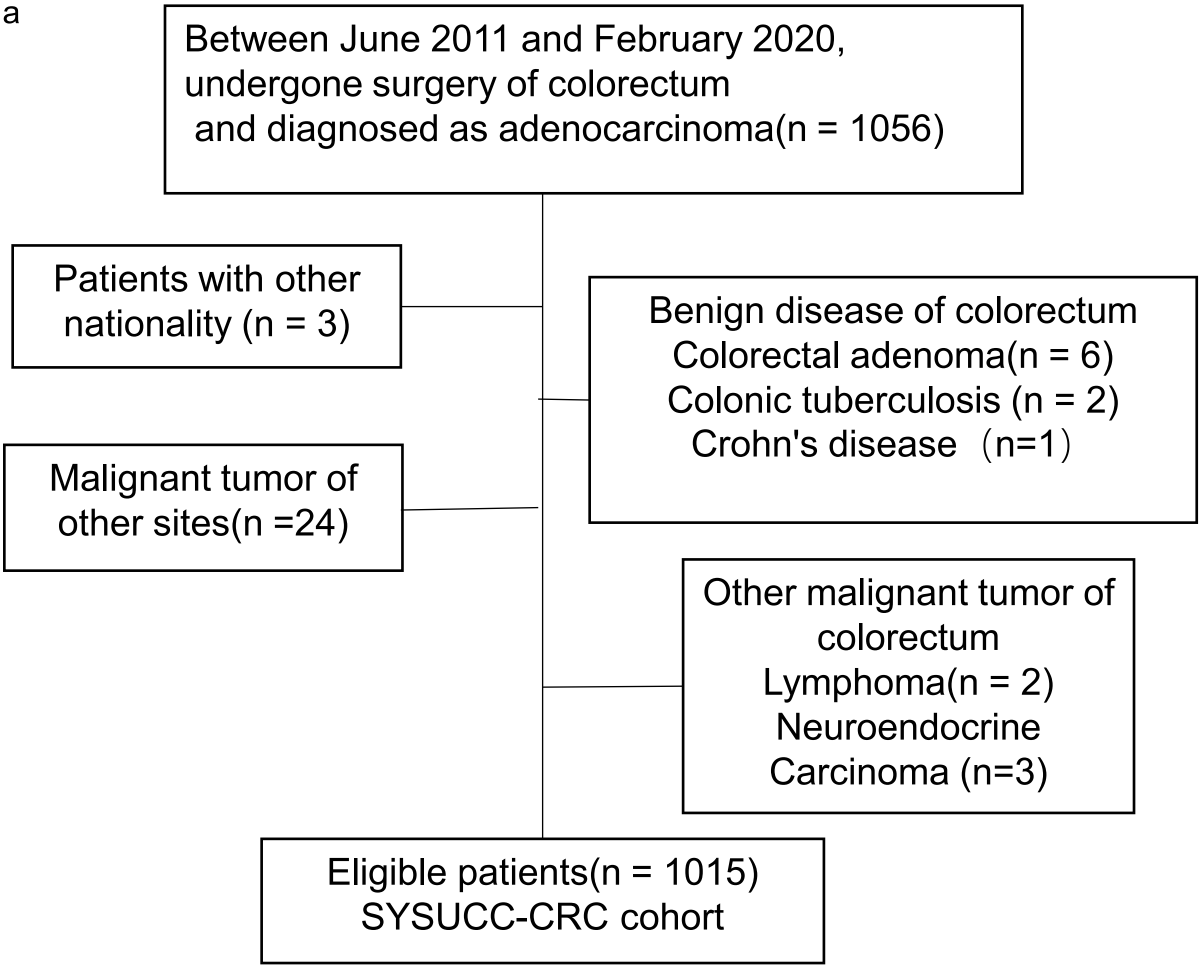

b

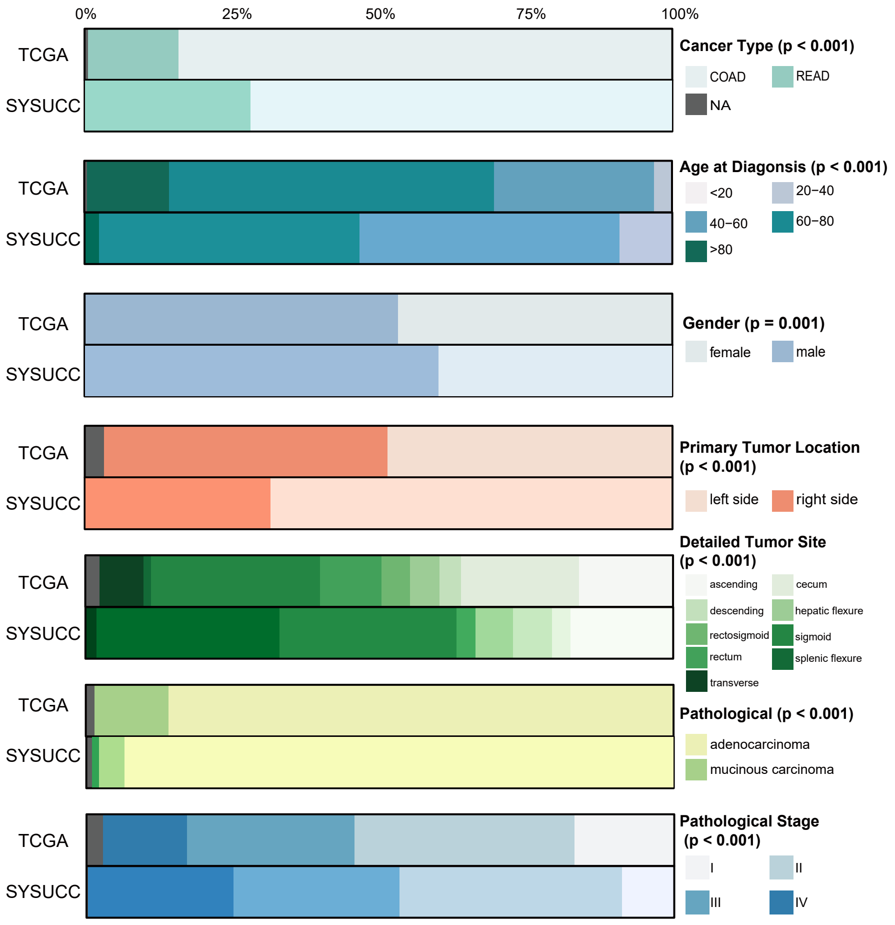

Supplementary Figure 1. Data collation and clinical data of Sun Yat-Sen University Cancer Center colorectal cancer (SYSUCC-CRC) cohort. **a.** Flowchart of data collation for SYSUCC-Cohort. **b.** Comparison between SYSUCC-CRC cohort and TCGA-CRC cohort in terms of cancer type, age at diagnosis, gender, primary tumor location, detailed tumor site, pathological type and pathological stage.

Supplementary Figure 2

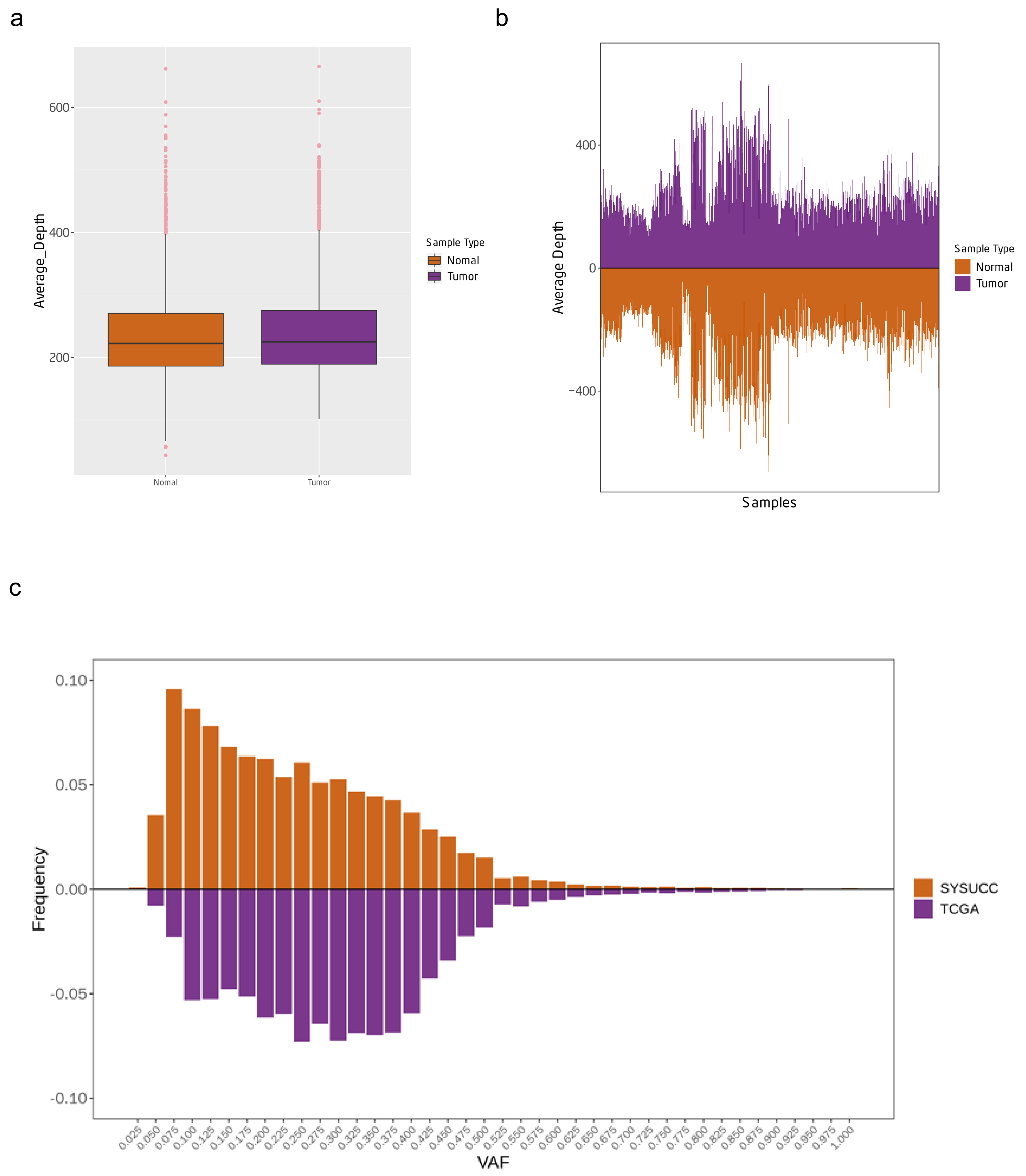

Supplementary Figure 2. Sequencing depth and variant allele frequency (VAF) of the whole exome sequencing data in SYSUCC-CRC cohort. **a.** Average **s**equencing depth in tumors and normal tissues. (horizontal lines inside the box represent the median; the top ends of the box represent the lower quartile; the bottom ends of the box represent the lower quartile; the whiskers above the box plot extend from the upper quartile to the highest actual value that is within the (75th percentile + 1.5 * (interquartile range));the whiskers below the box plot extend from the lower quartile to the lowest actual value that is within the (25th percentile - 1.5 * (interquartile range))). **b.** Paired comparison of average sequencing depth between tumors (n = 1015) and normal tissues (n = 1015). **c.** Frequency distribution of variant allele frequency (VAF) in SYSUCC-CRC cohort and TCGA-CRC cohort.

Supplementary Figure 3

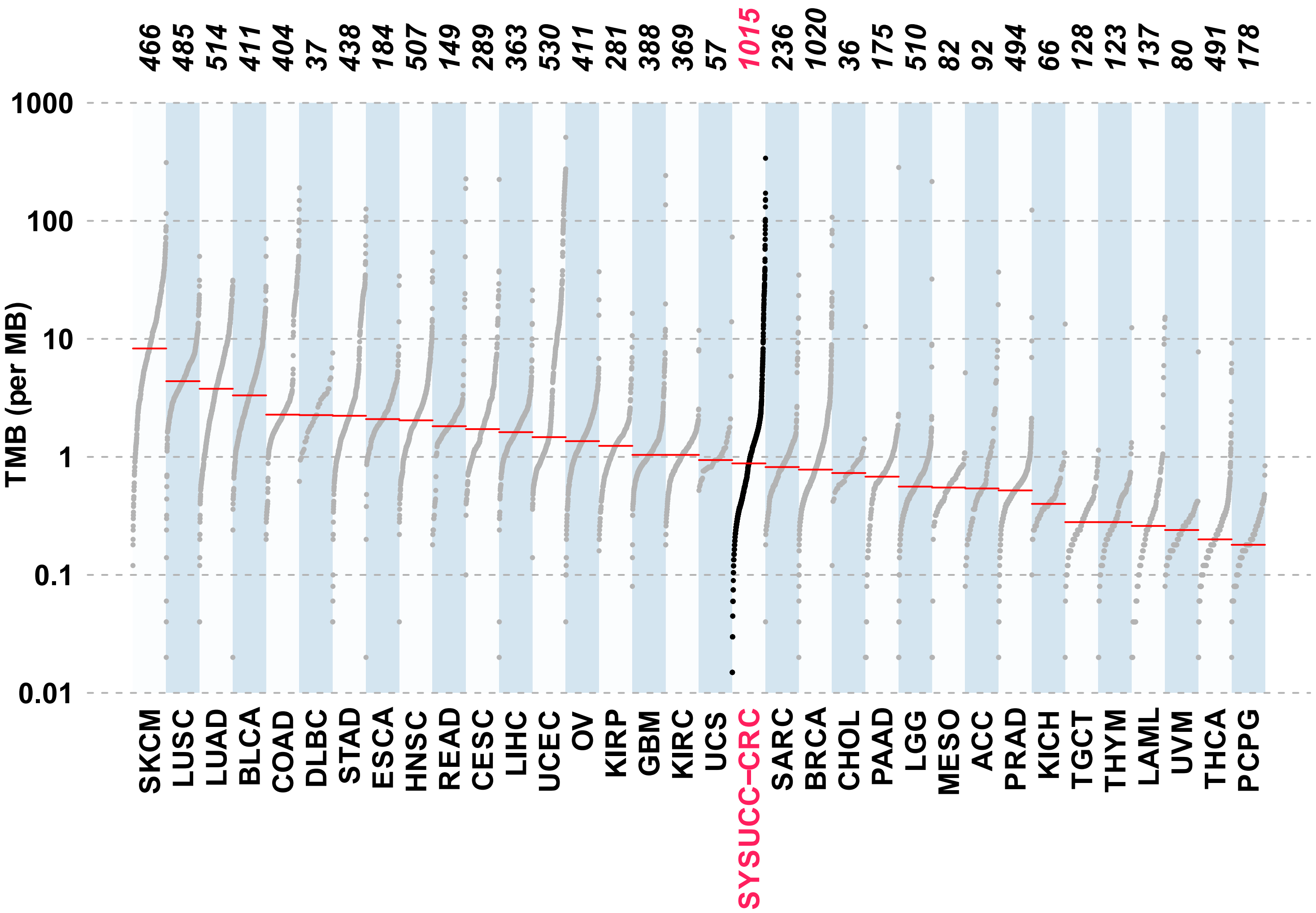

Supplementary Figure 3. Tumor mutation burden in SYSUCC-CRC cohort and TCGA pan cancer cohorts.

Supplementary Figure 4

a

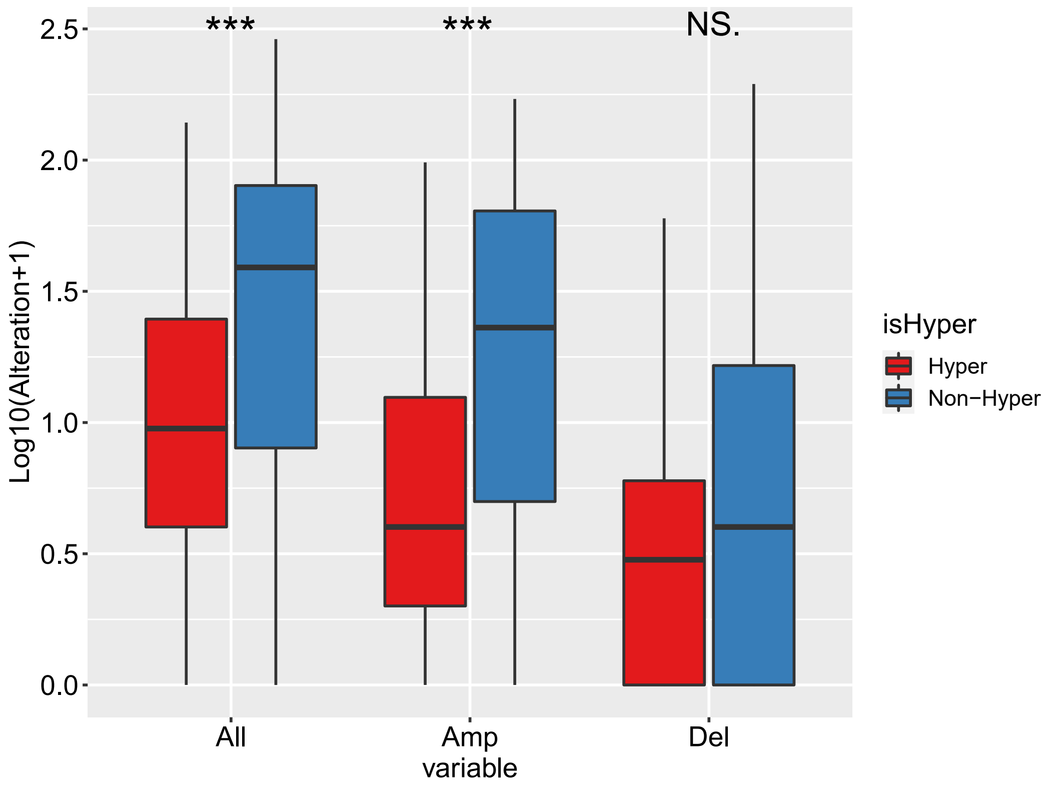

b

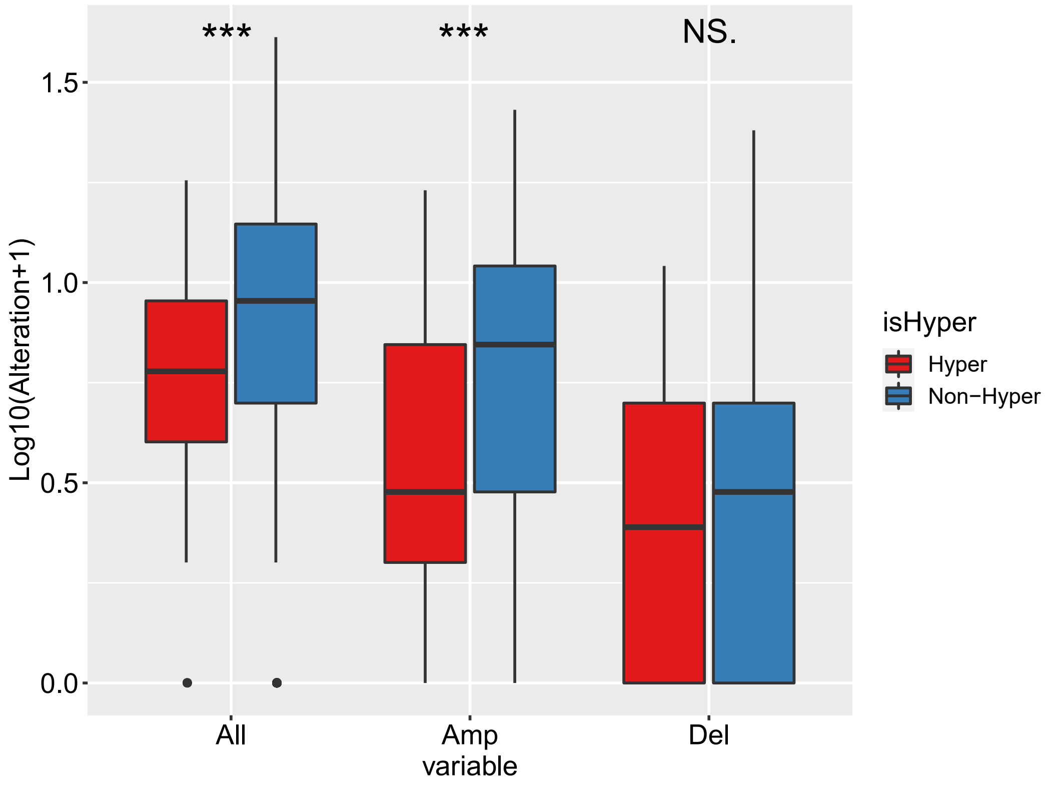

Supplementary Figure 4. Comparison of tumor copy number variation burden between hypermutated CRC samples (n = 72) and non-hypermutated CRC samples (n = 943). **a.** Comparison of tumor copy number variation burden on gene level between hypermutated CRC samples and non-hypermutated CRC samples. **b.** Comparison of tumor copy number variation burden on lesion level between hypermutated CRC samples and non-hypermutated CRC samples. (Wilcox Rank-Sum Test (two-sided, no multiple adjustment): NS. means P-value; **P-value* < 0.05; ***P-value* < 0.01; ****P-value* < 0.001) (horizontal lines inside the box represent the median; the top ends of the box represent the lower quartile; the bottom ends of the box represent the lower quartile; the whiskers above the box plot extend from the upper quartile to the highest actual value that is within the (75th percentile + 1.5 * (interquartile range));the whiskers below the box plot extend from the lower quartile to the lowest actual value that is within the (25th percentile - 1.5 * (interquartile range)))

Supplementary Figure 5

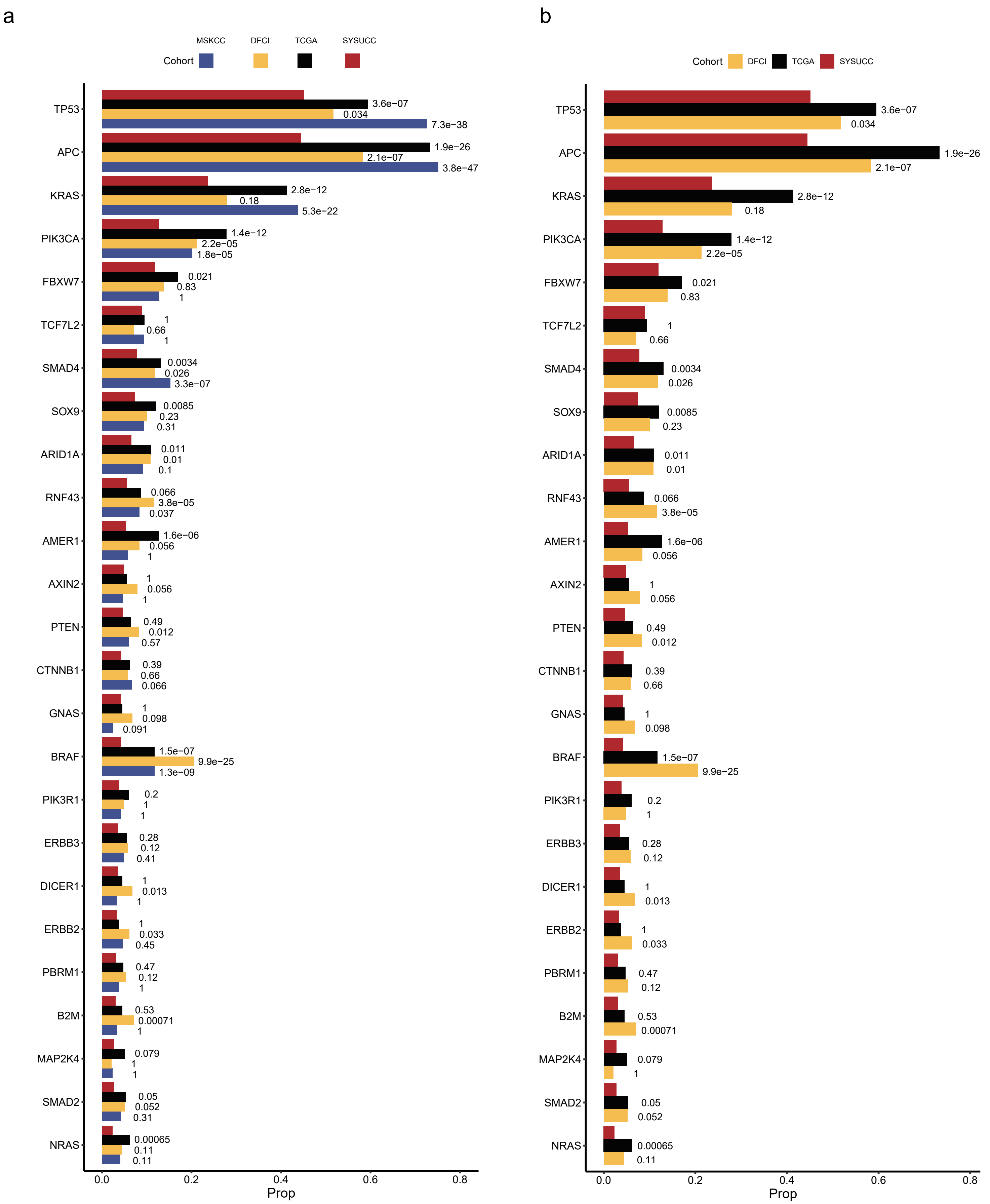

Supplementary Figure 5. Mutation frequency of significantly mutated genes across SYSUCC-CRC cohort (n = 1015), DFCI-CRC cohort (n = 619), MSKCC-CRC cohort (n = 1134) and TCGA-CRC cohort (n = 594). **a.** Significantly mutated genes involved in MSK-IMPACT panel. **b.** Significantly mutated genes not involved in MSK-IMPACT panel. P value of the comparison between a specific cohort and SYSUCC-CRC cohort was presented following the bar of corresponding cohorts (Chi-square Test (two-sided, bonferroni multiple adjustment within a gene).

Supplementary Figure 6

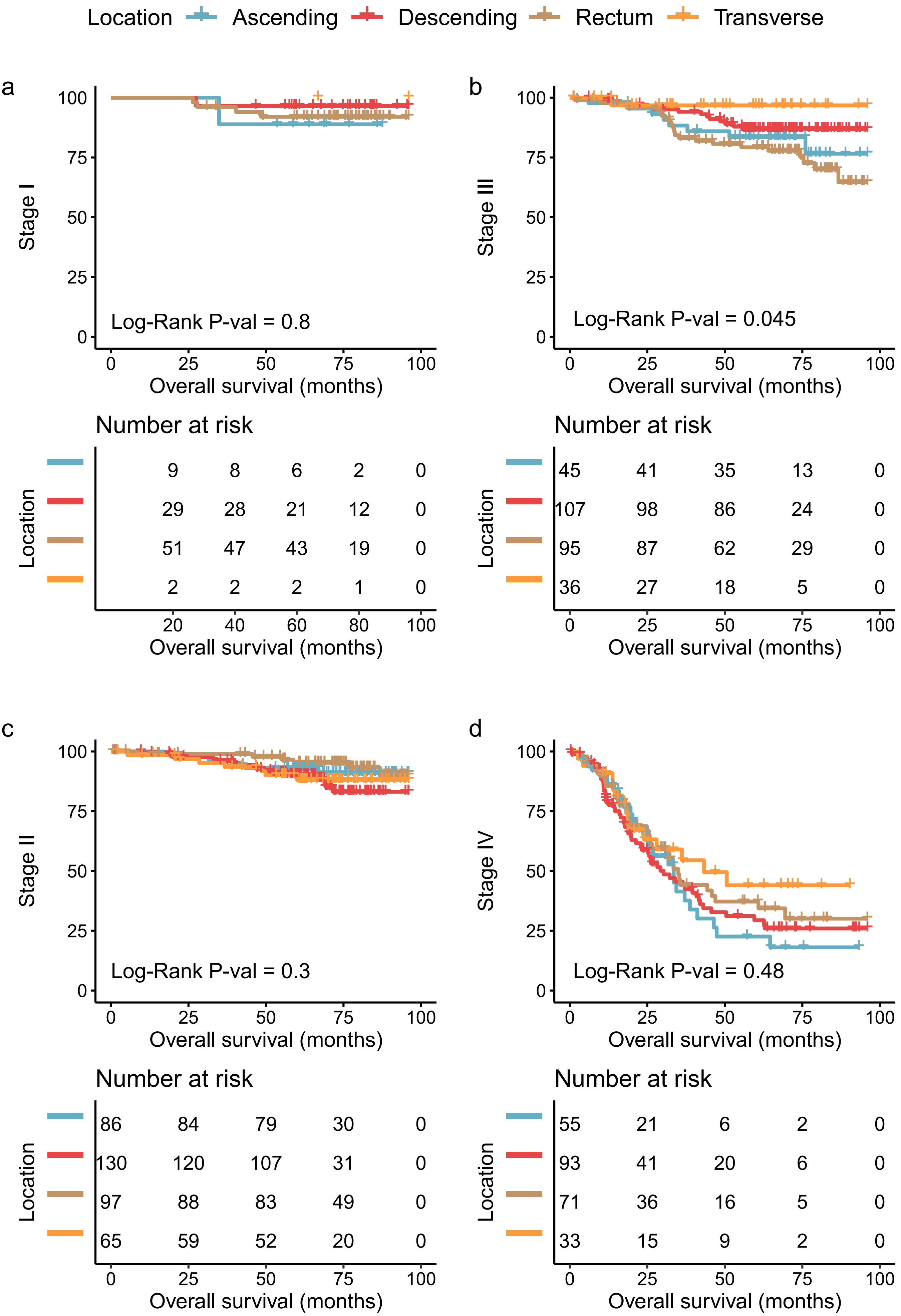

Supplementary Figure 6. Kaplan-Meier estimates of overall survival (OS) comparing the CRC patients with different primary tumor locations in different pathological stages. **a.** Kaplan-Meier estimates of overall survival (OS) comparing the CRC patients with different primary tumor locations in stage I. **b.** Kaplan-Meier estimates of OS comparing the CRC patients with different primary tumor locations in stage II. **c.** Kaplan-Meier estimates of OS comparing the CRC patients with different primary tumor locations in stage III. **d.** Kaplan-Meier estimates of OS comparing the CRC patients with different primary tumor locations in stage IV.

Supplementary Figure 7

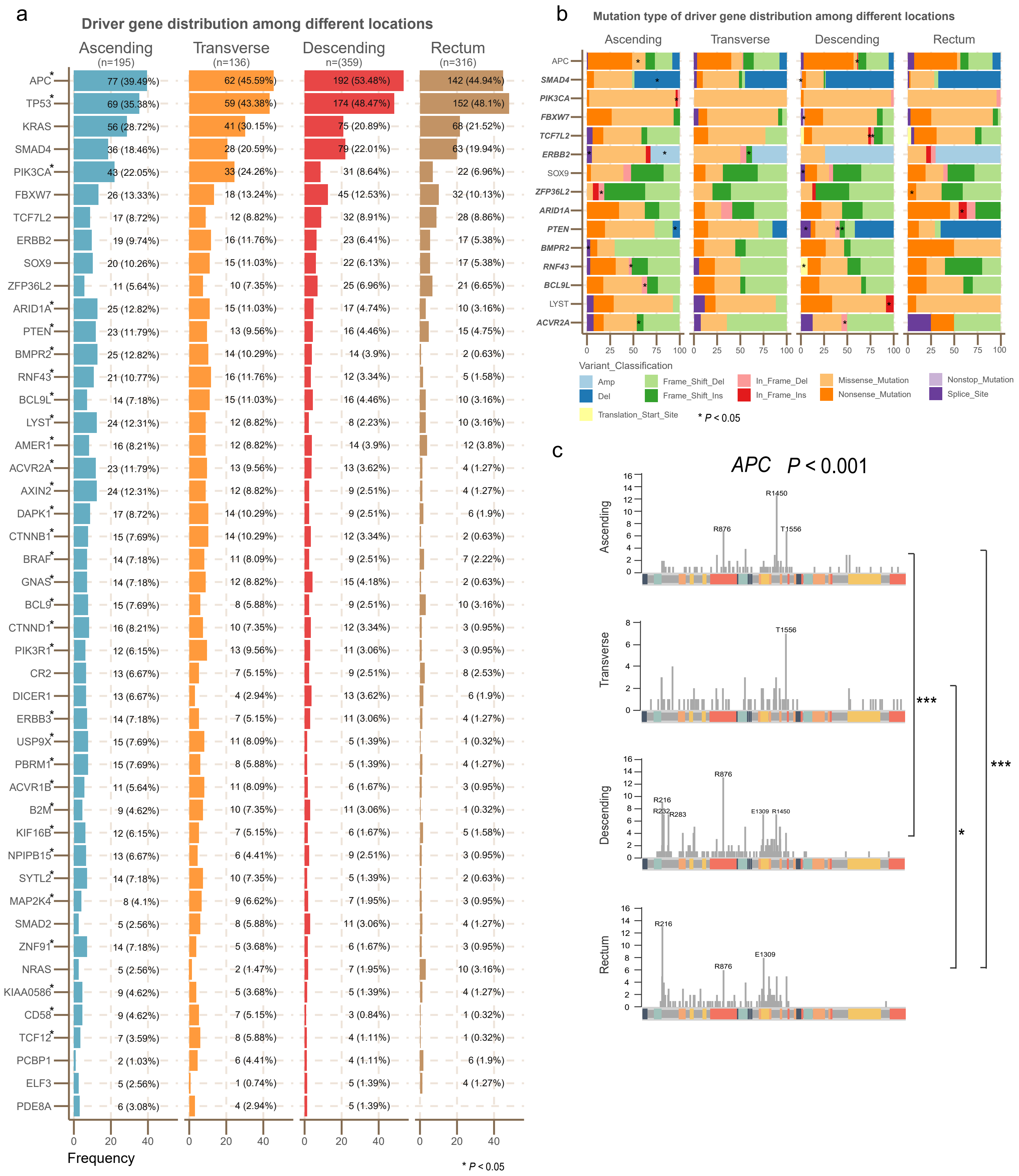

Supplementary Figure 7. Comparison of mutational characteristics between CRCs in different locations. **a.** Bar plot depicting mutant counts and frequencies of HC-SMG genes among different colorectal tumor locations. The X-axis represents the gene mutation frequency according to tumor location. Chi-squared test (two-sided, no multiple adjustment), *P < 0.05. **b.** Bar plot showing the percentage of mutation type of each gene in different locations. Asterisks indicate that the mutation type frequency is significantly different among locations. Chi-squared test (two-sided, no multiple adjustment), *P < 0.05. **c.** Hotspot distributions of *APC* among locations. One-way analysis of variance (ANOVA) was used to determine whether there were any statistically significant differences between the four locations. A post hoc Tukey HSD test was used to perform multiple comparisons of location pairs (two-sided). *P < 0.05. **P < 0.01. ***P < 0.001. Only statistically significant comparison was denoted on the plot.

Supplementary Figure 8

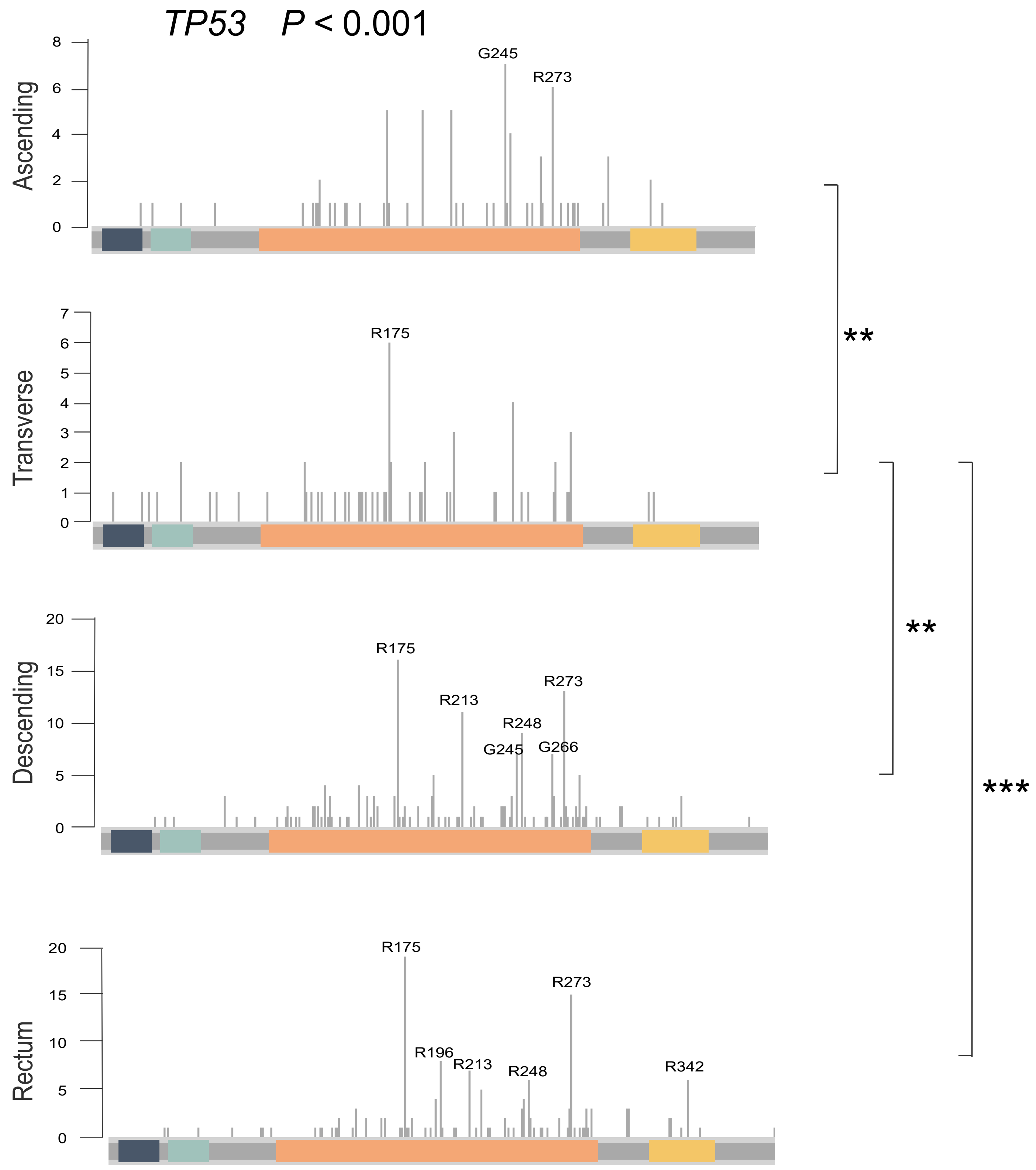

Supplementary Figure 8. Locational distribution of somatic TP53 mutation in TP53-mutated CRCs with different primary location. Statistical significance between the groups was denote by “*” in the right side of the plot (Tukey HSD test, two-sided, **P-value* < 0.05; ***P-value* < 0.01; ****P-value* < 0.001, Only statistically significant comparison was denoted on the plot). Hotspot mutations in TP53 were annotated with the referent amino acid and its location.

Supplementary Figure 9

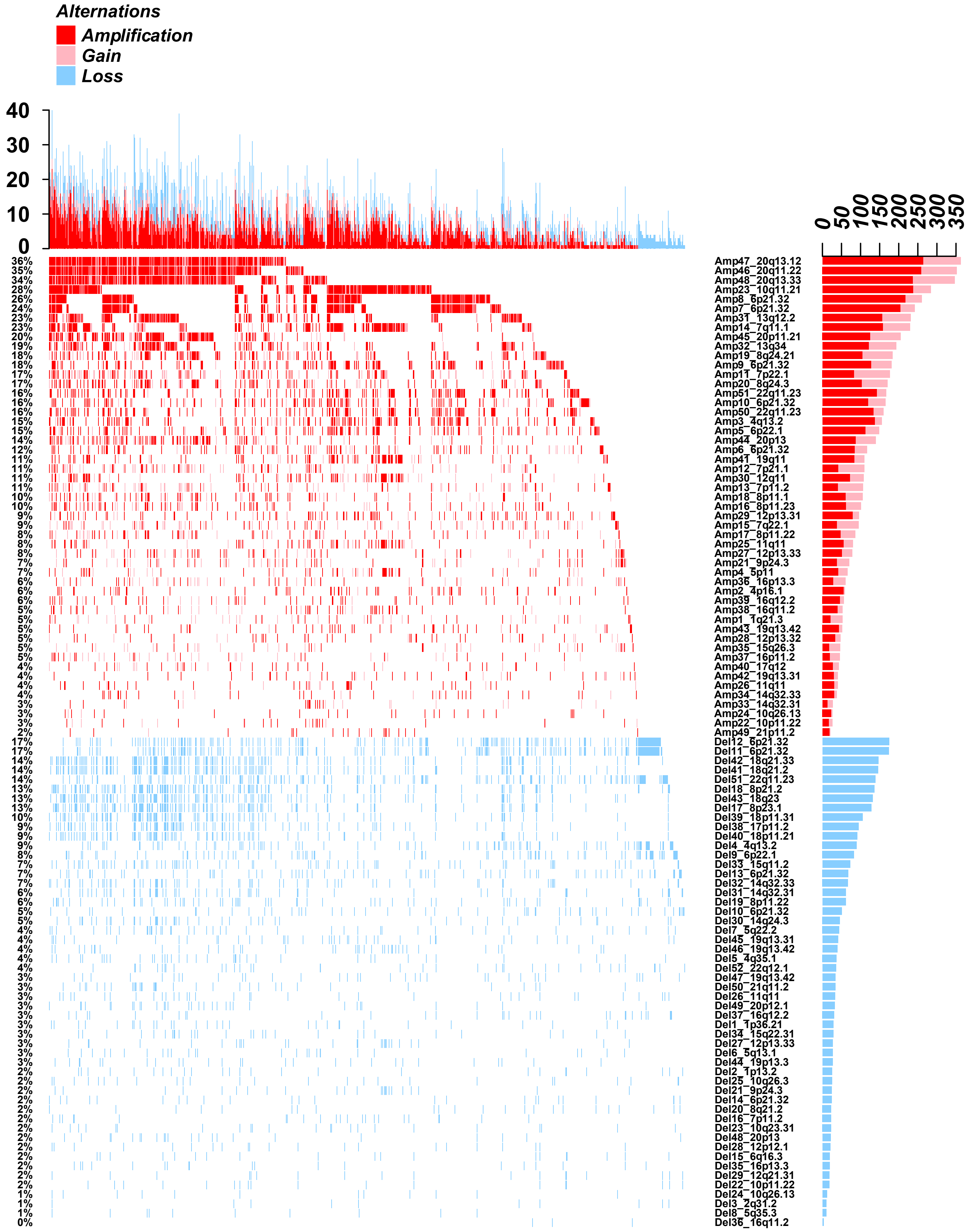

Supplementary Figure 9. Copy number variation at focal level in SYSUCC-CRC cohort. **Top:** Bars represent copy number variation burden for the CRC samples with different variation types distinguished by color (red for amplifications and blue for deletions). **Bottom:** significant CNV lesion in SYSUCC-CRC cohort ranked by variation frequency. Variation color indicates the class of CNV (red for amplifications and blue for deletions).

Supplementary Figure 10

a

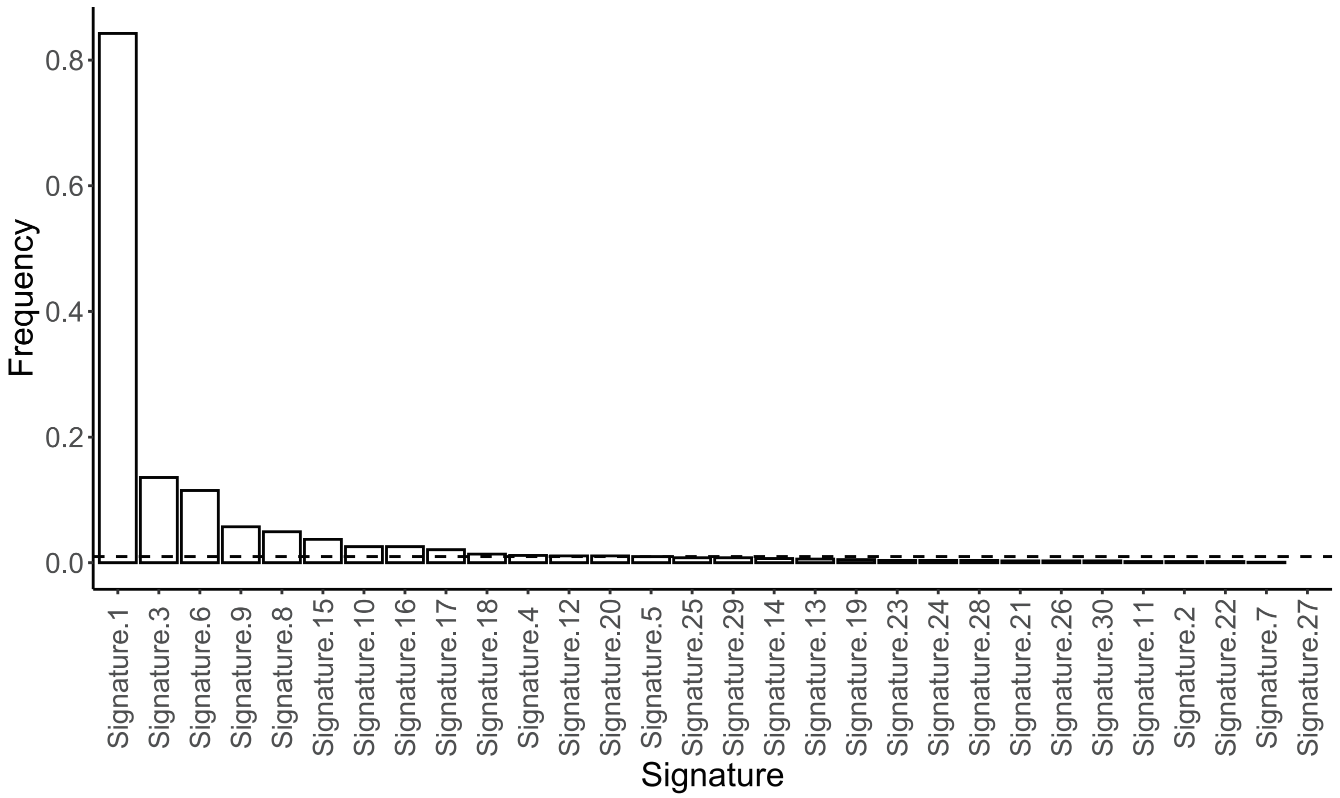

b

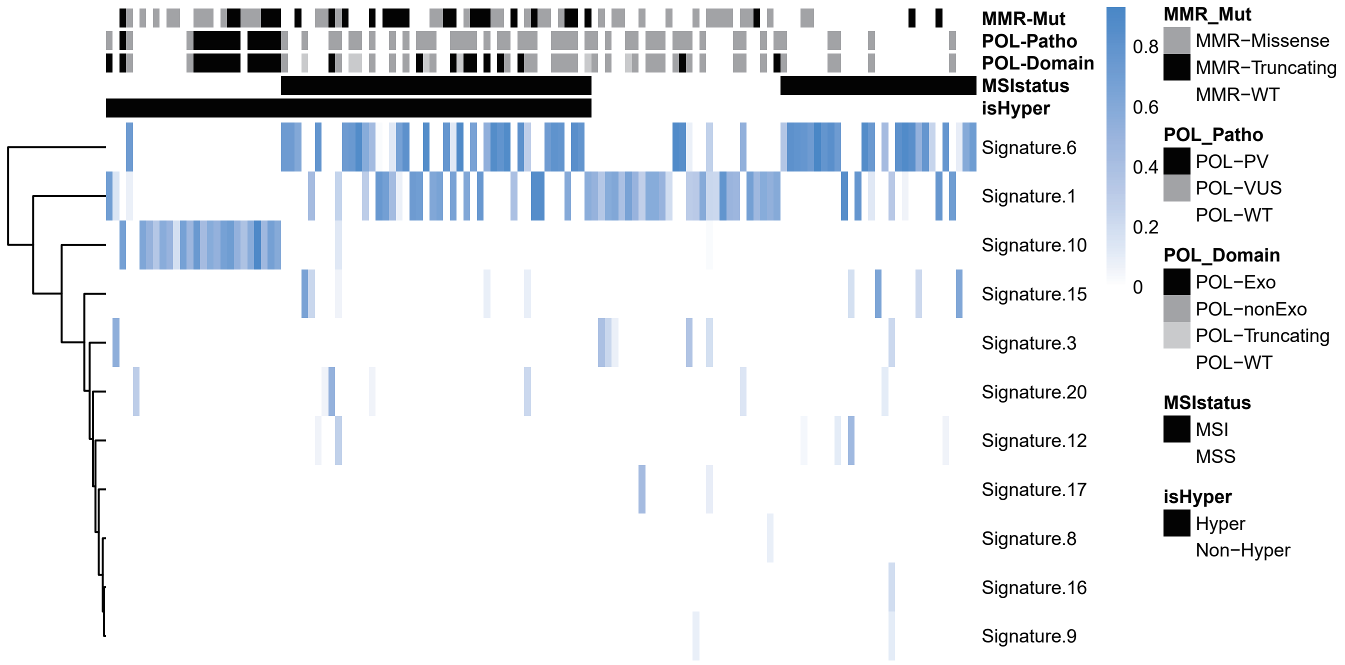

Supplementary Figure 10. Detection frequency of each mutation signature and their distribution in hypermutated CRC samples. **a.** Detection frequency of each single base substitution (SBS) signature in SYSUCC-CRC cohort. 1% (dashed line in the plot) was set as the cutoff to define the recurrent mutation signature in the cohort. **b.** Recurrent mutation signature in hypermutated CRC subgroup. **Top:** Sample annotation including mutation status of mismatch repair (MMR) genes, mutation status of POLE/POLD1 annotated with OncoKB database (POL-PV for POLE/POLD1 pathological variation, POL-VUS for POLE/POLD1 variants of unknown significance), mutation status of POLE/POLD1 annotated by domanial location (POL-Exo for POLE/POLD1 missense mutation occurring in exonuclease domain, POL-nonExo for POLE/POLD1 missense mutation occurring out of exonuclease domain, POL-Truncating for POLE/POLD1 truncating mutation), MSI status and hypermutated status. Bottom: frequency of each detected signature in the hypermutated CRC samples.

Supplementary Figure 11

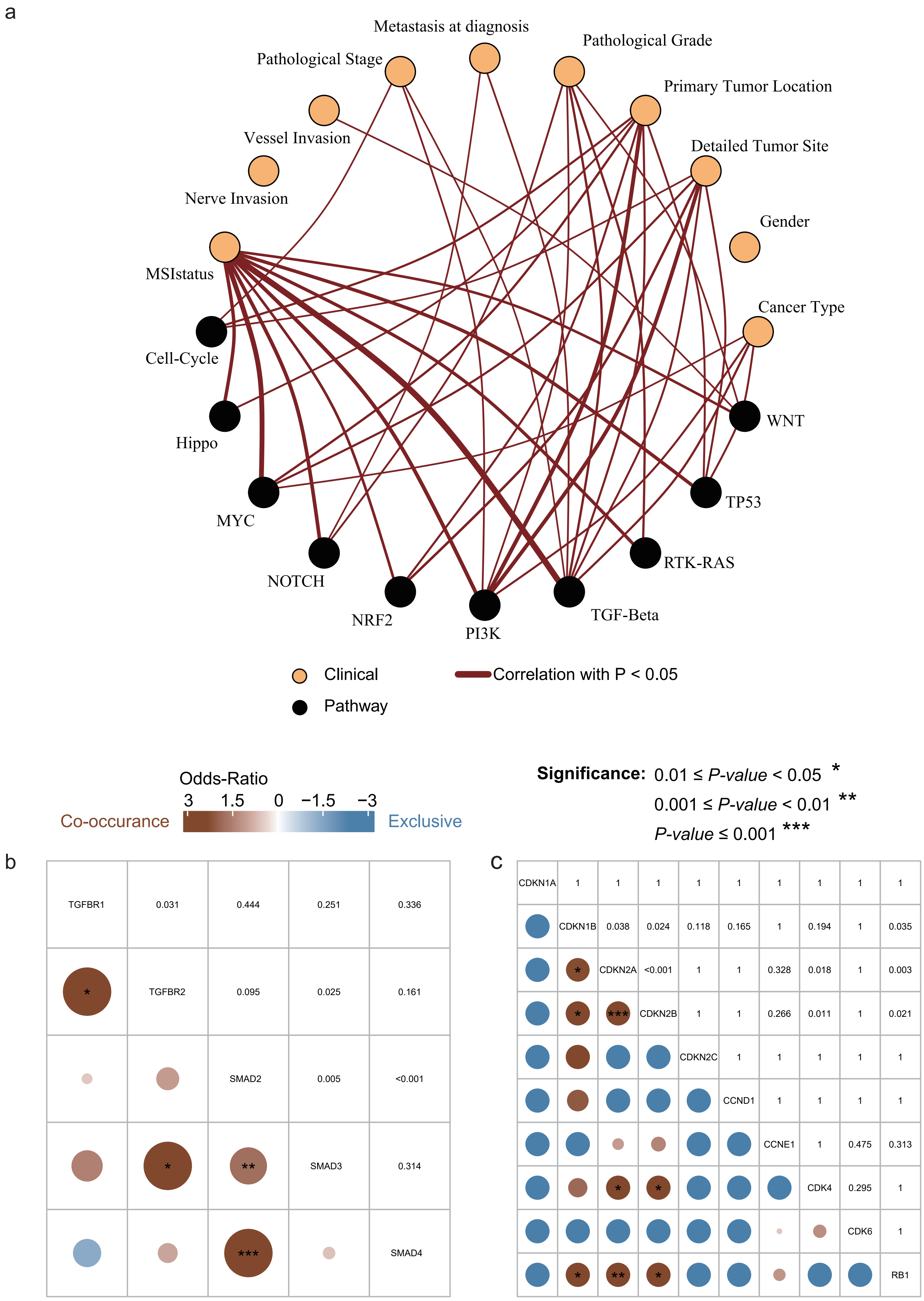

Supplementary Figure 11. Correlation between clinical features and alteration of oncogenic pathways. **a.** Network plot presented the correlation analysis of the clinical features and alteration of oncogenic pathway. The size of the line indicates -log(P-value) calculated by correlation test. **b.** Concurrent and exclusive analysis on oncogenic alteration of the genes in Cell-Cycle pathway. **c.** Concurrent and exclusive analysis on oncogenic alteration of the genes in TGF-beta pathway. Color and size of the circle denote the extend of the oncogenic alteration relationship (Odds Ratio) of the two genes respectively. Significant symbol in the circle and the number in the frame denote the significance of the oncogenic alteration relationship of the two genes respectively (Chi-square test, two-sided, *P<0.05, **P<0.01).

Supplementary Figure 12

a

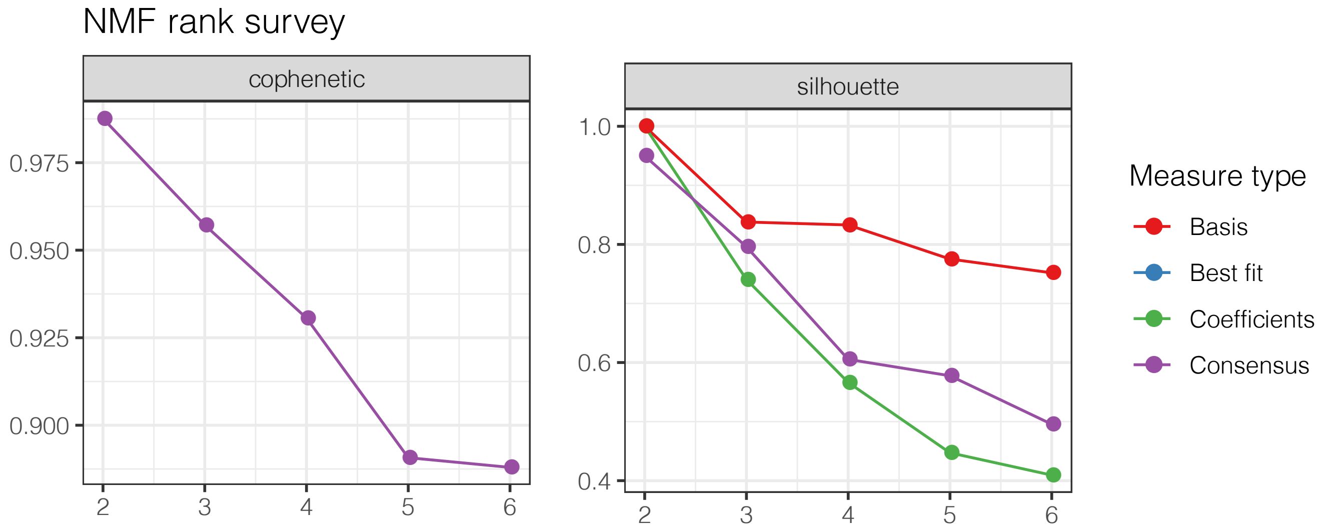

b

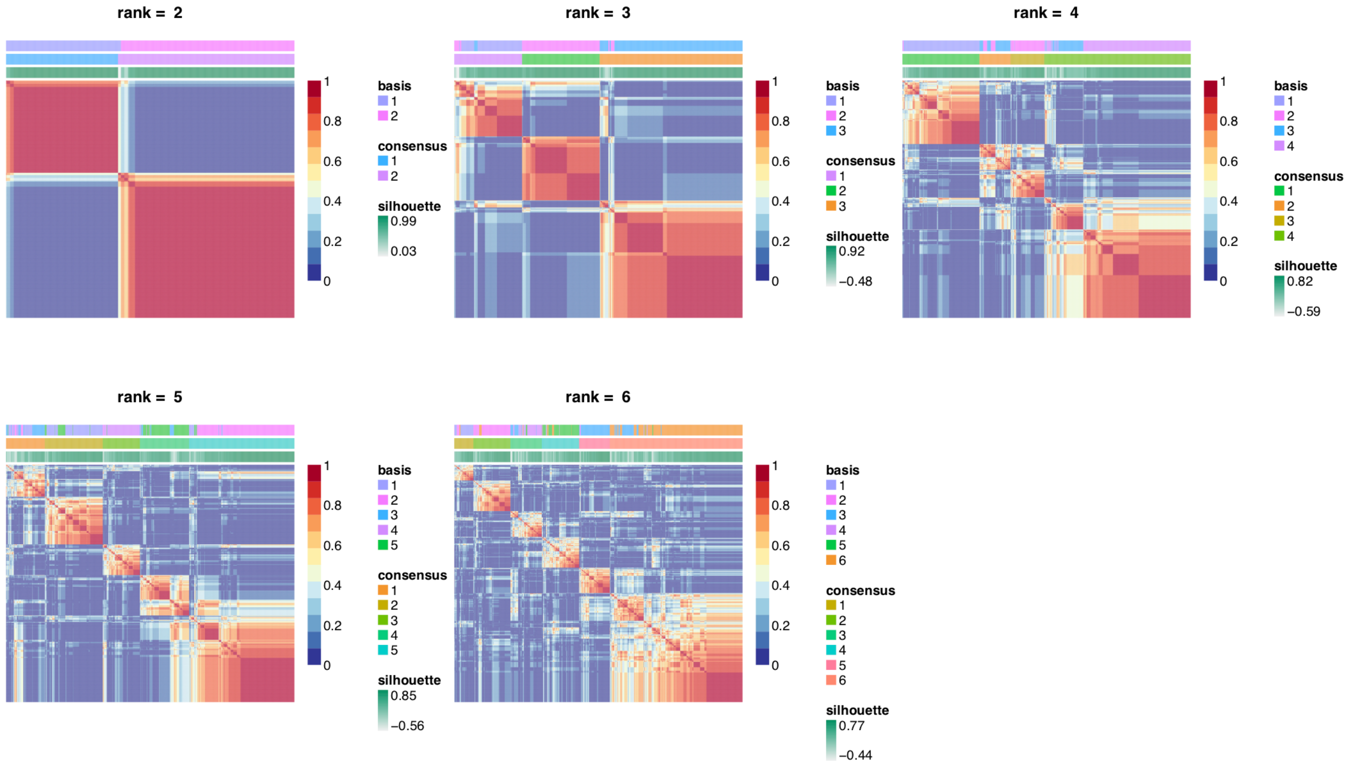

Supplementary Figure 12. Consensus non-negative matrix factorization (NMF) cluster for SYSUCC-CRCs. **a.** Cophenetic plot and silhouette of ranks ranging from 2 to 6. **b.** Consensus cluster heatmap of ranks ranging from 2 to 6.

Supplementary Figure 13

a

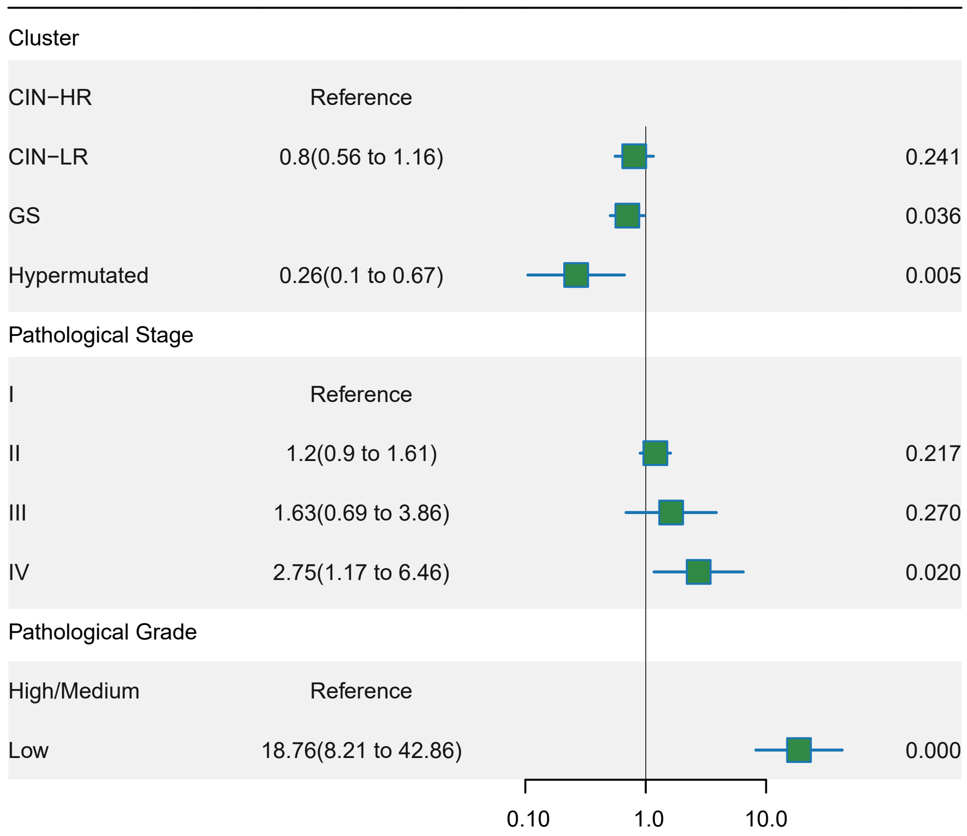

b

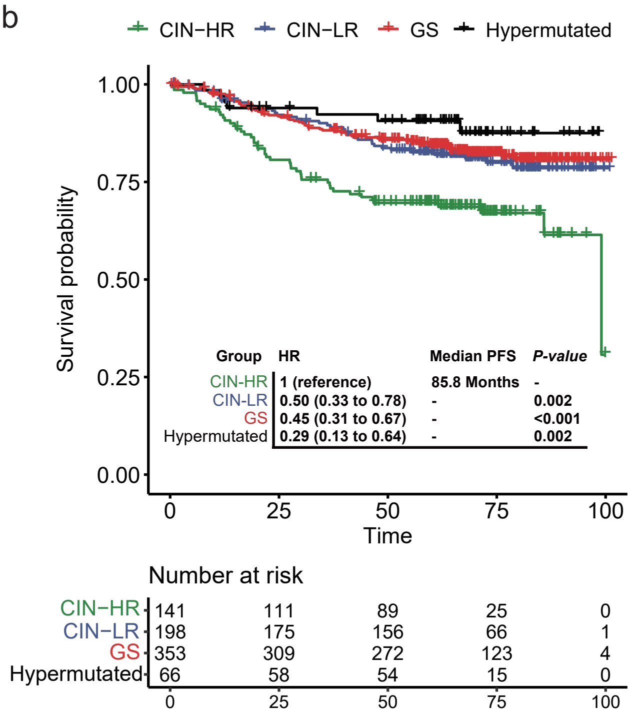

c

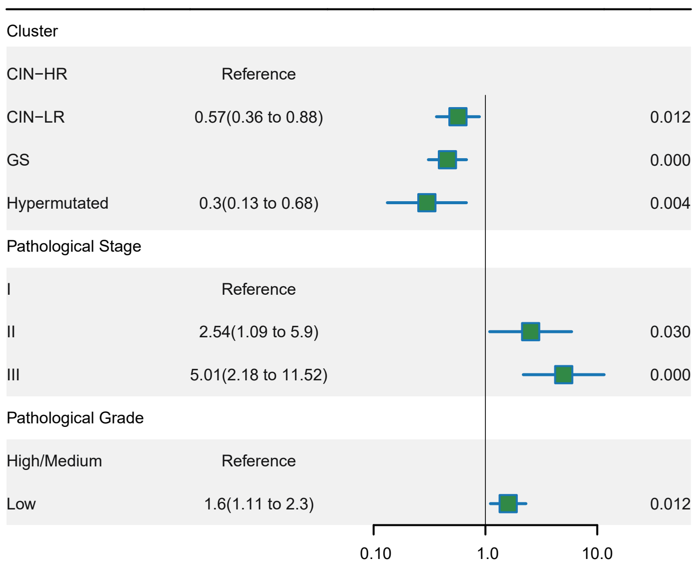

Supplementary Figure 13. Clinical relevance of genomic subtypes of CRC in Chinese patients. **a.** Forrest plot depicting the association between OS and genomic subtypes after correcting pathological stages and pathological grades. **b.** Kaplan-Meier estimates of disease-free survival (DFS) comparing the four CRC subtypes. **c.** Forrest plot depicting the association between DFS and genomic subtypes after correcting pathological stages and pathological grades.

Supplementary Figure 14

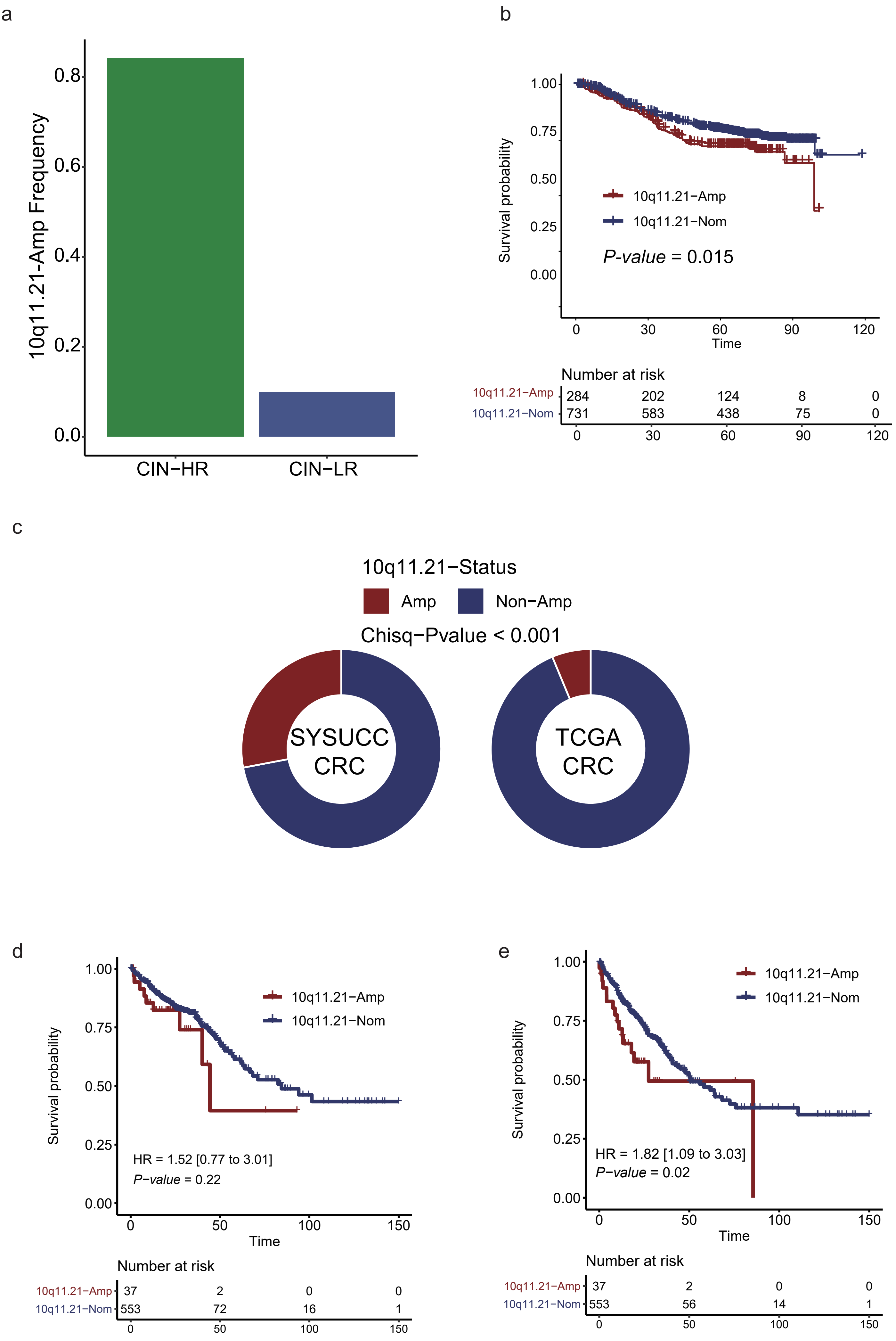

Supplementary Figure 14. Validation of the genomic subtypes in TCGA cohort. **a.** Frequency of 10q11.21 amplification in CIN-HR and CIN-LR. **b.** Association between OS and 10q11.21 amplification estimated by Kaplan-Meier method. **c.** Amplification frequency of 10q11.21 comparing CRC patients in SYSUCC-CRC cohort and those in TCGA-CRC cohort (Chi-square test, two-sided). **d.** Association between overall survival (OS) and 10q11.21 amplification in TCGA cohort. **e.** Association between progression-free survival (PFS) and 10q11.21 amplification in TCGA cohort.

Supplementary Figure 15

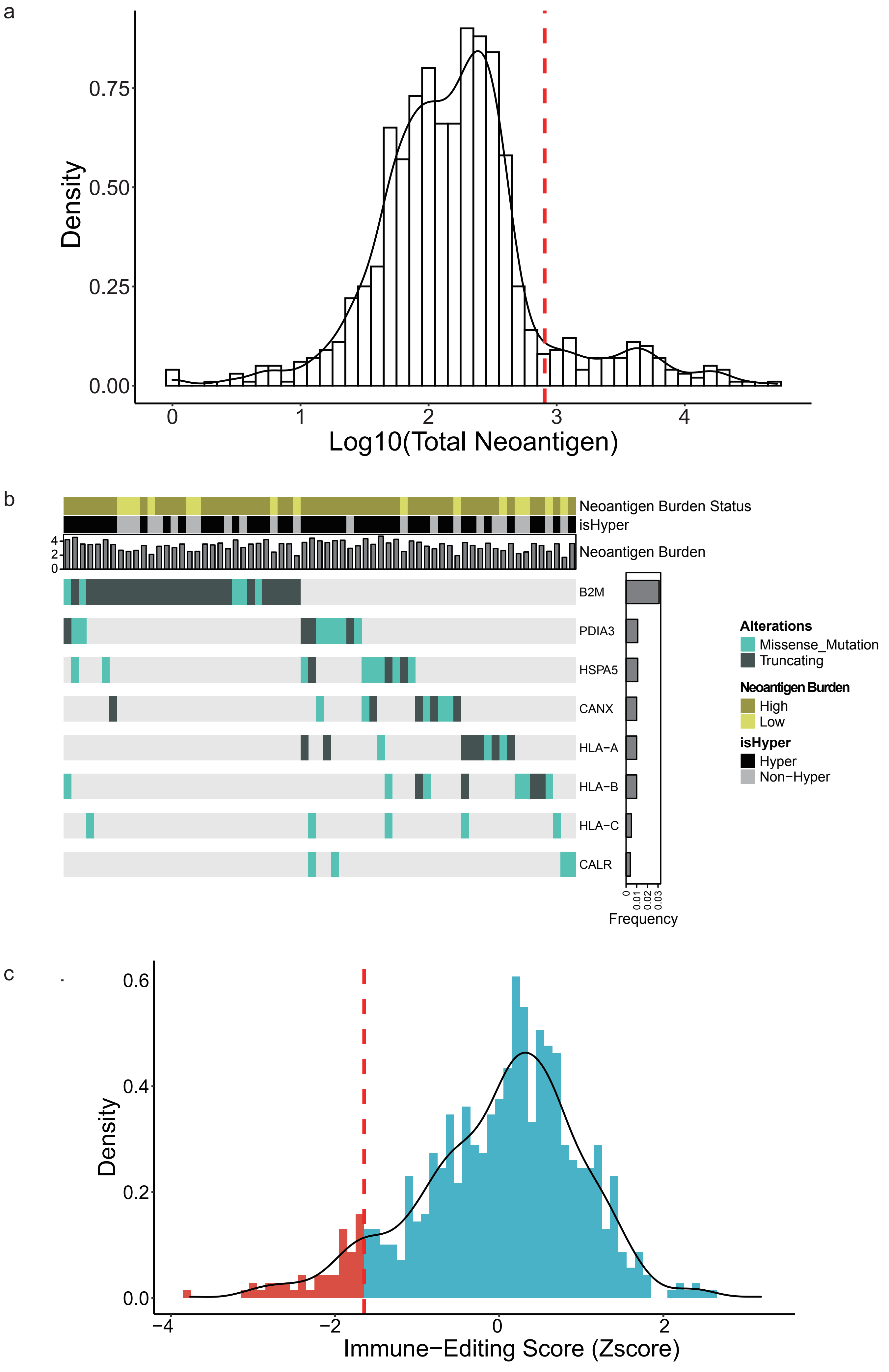

Supplementary Figure 15. Immunogenicity reducing was associated with poor prognosis in CRC patients carrying low neo-antigen burden. **a.** Density distribution of total neoantigen burden across CRC patients in SYSUCC cohort. (Red line denoted 90% quantile, which divided the patients into high neo-antigen burden (HNB) group and low neo-antigen burden (LNB) group) **b.** Oncoplot depicting the mutations of antigen presentation genes in SYSUCC-CRC cohort. **C.** Density distribution of immune-editing score after Z-score conversion (the vertical line (x = -1.645) divided the patients into immune-editing group (red) and non-immune-editing group).

Supplementary Figure 16

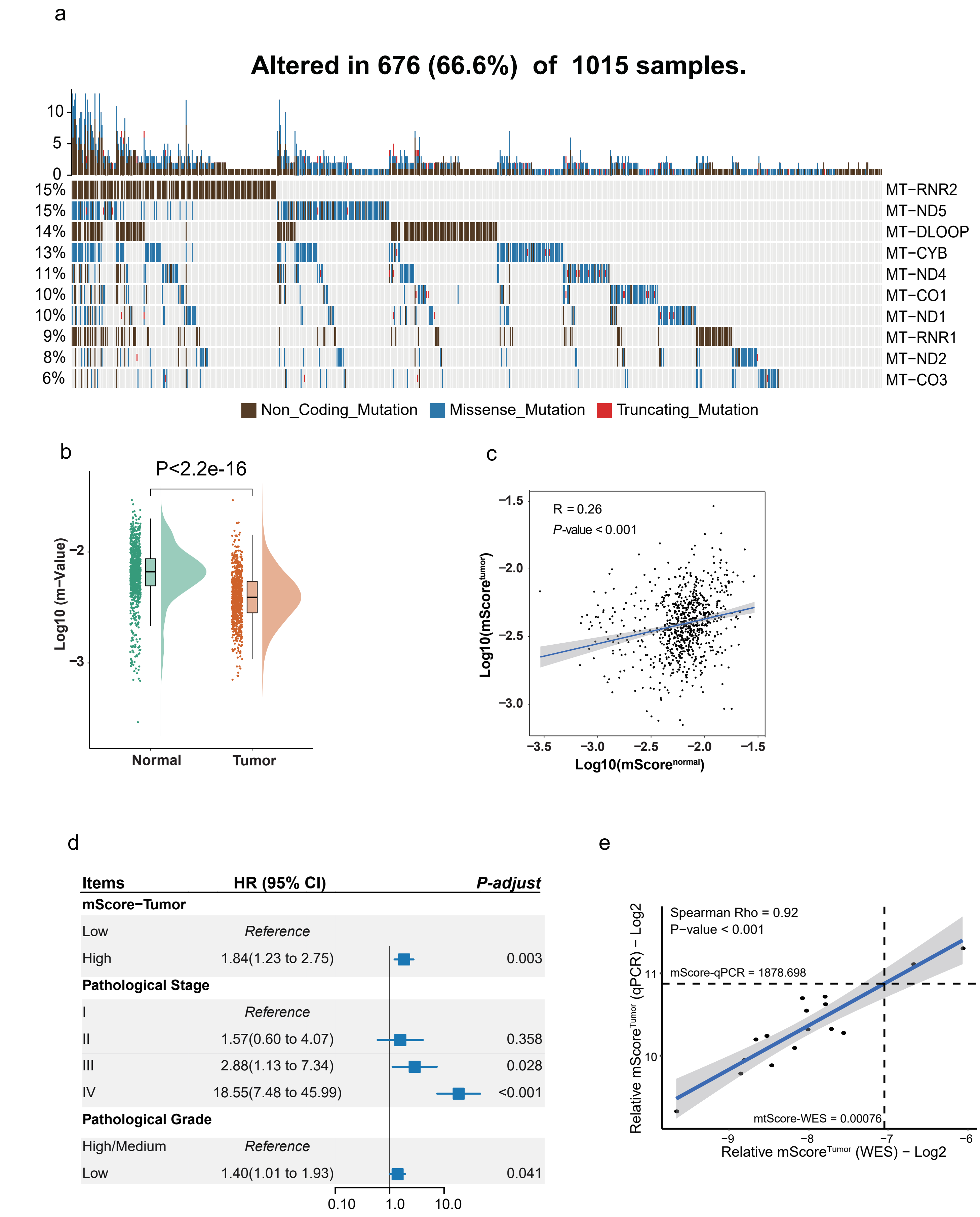

Supplementary Figure 16. Characteristics of the mitochondrial genome in Chinese CRC patients. **a.** Top: Bars represent somatic mitochondrial gene mutation rates for CRC samples with different mutation types distinguished by color. Bottom: Mutated mitochondrial genes in Chinese CRC patients ranked by mutation frequency. Mutation color indicates the class of mutation. **b.** Comparison of the relative copy number of mtDNA (denoted by the mScore) in tumor and normal tissues (Wilcoxon rank-sum test, two sided).(horizontal lines inside the box represent the median; the top ends of the box represent the lower quartile; the bottom ends of the box represent the lower quartile; the whiskers above the box plot extend from the upper quartile to the highest actual value that is within the (75th percentile + 1.5 * (interquartile range));the whiskers below the box plot extend from the lower quartile to the lowest actual value that is within the (25th percentile - 1.5 * (interquartile range))). **c.** Relationship between the mScore in tumor and normal tissues and the corresponding linear regression model construction (shadow around the line denotes the 95% interval). **d.** Forrest plot depicting the association between OS and mScore status after correcting pathological stages and pathological grades. **e.** Correlation between relative $mScore^{Tumor}$ quantified by WES and relative $mScore^{Tumor}$ quantified by qPCR. Linear regression model was used to define the cutoff of $mScore^{Tumor}$ quantified by qPCR according to the pre-set cutoff from $mScore^{Tumor}$ quantified by WES (shadow around the line denotes the 95% interval).

Supplementary Figure 17

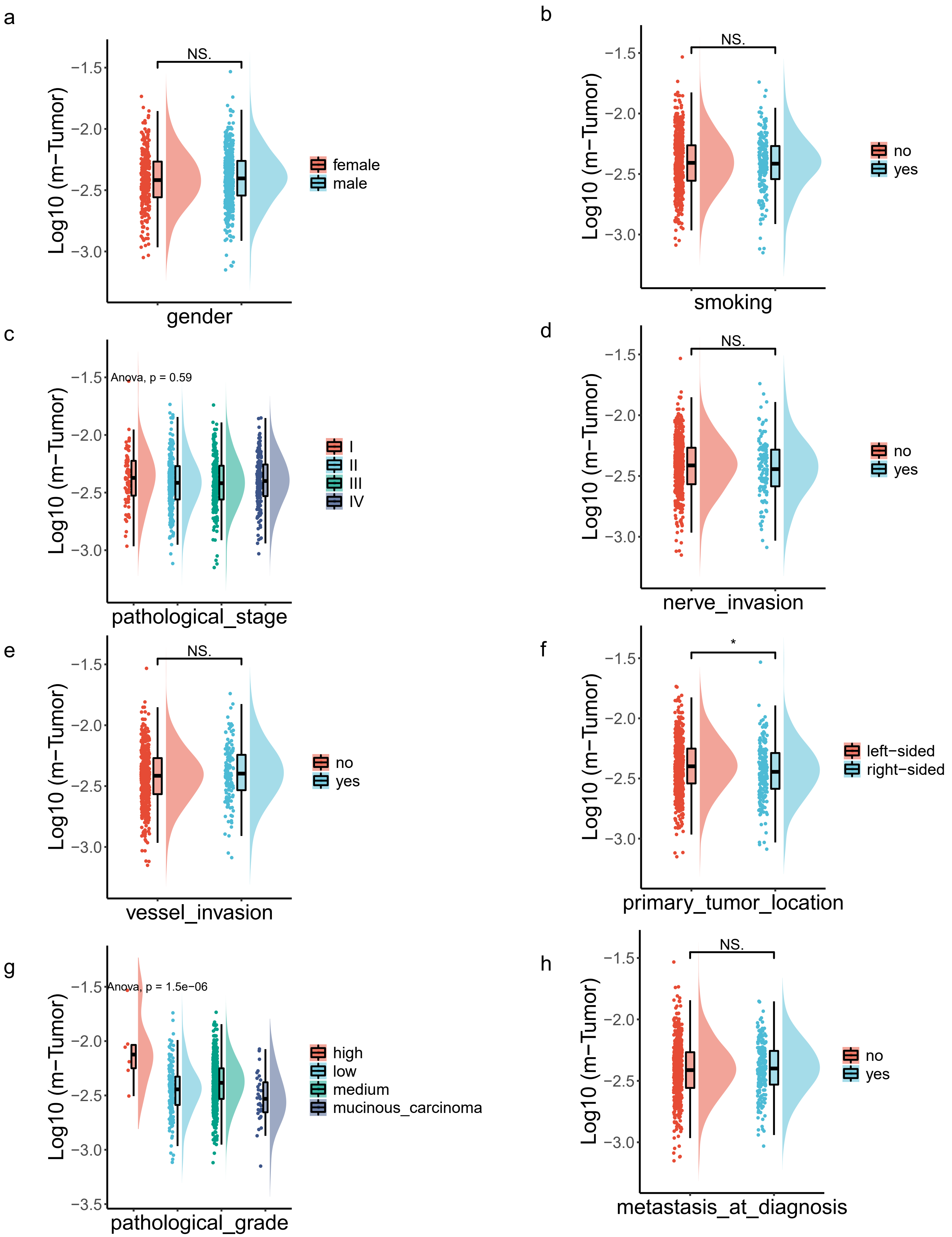

Supplementary Figure 17. Clinical relevance of relative copy number of mitochondria DNA denoted by mScore (except C&G, all statistical tests were performed by Wilcoxon rank-sum test, two sided; NS. means P-value >= 0.05; **P-value* < 0.05; ***P-value* < 0.01; ****P-value* < 0.001). (horizontal lines inside the box represent the median; the top ends of the box represent the lower quartile; the bottom ends of the box represent the lower quartile; the whiskers above the box plot extend from the upper quartile to the highest actual value that is within the (75th percentile + 1.5 * (interquartile range));the whiskers below the box plot extend from the lower quartile to the lowest actual value that is within the (25th percentile - 1.5 * (interquartile range)))
